# A review of *Acromastigum* (Lepidoziaceae, Marchantiophyta) in East Indochina

**DOI:** 10.1371/journal.pone.0356719

**Published:** 2026-09-03

**Authors:** Vadim A. Bakalin, Yulia D. Maltseva, Ksenia G. Klimova, Seung Se Choi, Van Sinh Nguyen, Hung Manh Nguyen

**Affiliations:** 1 Laboratory of Cryptogamic Biota, Botanical Garden-Institute FEB RAS, Vladivostok, Russia; 2 Team of National Ecosystem Survey, National Institute of Ecology, Seocheon, Republic of Korea; 3 Institute of Biology, Graduate University of Science and Technology, Vietnam Academy of Science and Technology, Ha Noi, Vietnam; Institute for Biological Research, University of Belgrade, SERBIA

## Abstract

*Acromastigum* A. Evans is revised in East Indochina. The distribution of six species is documented, including one species whose occurrence is not supported by available materials. One new-to-science species from the species-poor subgenus *Acromastigum* is described from the Lâm Viên Plateau of the Central Highlands of Vietnam. The report of *Acromastigum curtilobum* for Thailand is based on incorrect identification and should be referred to *A. herzogii*. *Acromastigum echinatum* and *A. laevigatum* are observed for the first time in Cambodia. A large number of new localities of *A. herzogii* have been found in Vietnam, where the species distribution extends from North Vietnam through the Annamite Range to the Lâm Viên Plateau. This species is the most common representative of the genus in East Indochina. Morphological and molecular data reveal a more natural relationship of *A. herzogii* and *A. stellare* to subg. *Inaequilatera* than to subg. *Acromastigum*, as previously suggested. These two subgenera are also distinct in their rhizoid distribution, which was not noted in previous research. Overall, *Acromastigum* is an uncommon component of plant communities in East Indochina and has never been found with either sporophytes or fertilized archegonia, suggesting its high sensitivity to disturbances in forest communities.

## Introduction

The genus *Acromastigum* is not speciose genus within the family Lepidoziaceae (subfam. Bazzanioideae). According to the World Liverwort Checklist [1], with later additions by Thouvenot [2] and Renner and Wilson [3], the genus *Acromastigum* comprises 48 species distributed in subg. *Acromastigum* (8 species), subg. *Inaequilatera* (Schiffn.) Grolle (32 species) and the artificial group *Incertae sedis* (8 species). Very roughly, in appearance, most species of the genus usually resemble small *Bazzania* (*Bazzania bilobata* N. Kitag., *B. mayebarae* S. Hatt., etc.) but usually lack secondary pigmentation (thus being pale greenish yellowish to pale green), although there are a number of exceptions [4]. *Acromastigum* was originally described by Evans [5], who first identified a peculiar type of branching in the ventral geotropic flagella (now called *Acromastigum*-type branching) as a basic feature of the genus. The cited author provides a detailed description of such branching (l.c.: 99): “The peculiar branches of the new genus are the flagella. These are similar in appearance to those of *Bazzania*, except that they bear small but distinct leaves near the base; but their place of origin is very different. Instead of developing in the axils of the underleaves, each flagellum arises at one side of an underleaf; instead of being surrounded by an indistinct sheath, showing that it is endogenous in origin, it is naked at the base and is, therefore, exogenous in origin; the underleaf, finally, beside which a flagellum is situated, is much narrower than an ordinary underleaf.” This character is still the basis for separation from the morphologically similar *Bazzania*, although some species of the latter are very similar to *Acromastigum* in other morphological traits [6].

The main taxonomic diversity of the genus occurs in Malesia, Melanesia and Australasia, with small additions in surrounding regions (East Asia, South Asia, Southeast Asia, Polynesia, and Micronesia) and very few species in Latin America and South Africa [7–12]. In the areas immediately adjacent to East Indochina, one species is reported in Thailand, namely, *Acromastigum curtilobum* A. Evans [13,14], and two species are known in China, namely, *Acromastigum herzogii* Grolle (Guangxi Province) and *A. divaricatum* (Nees) A. Evans ex Reimers (unverified report, cf. [15]. In East Indochina itself, which is understood here as Laos, Cambodia, and Vietnam, six species have been reported. These initial data are described below. Our recent collections in East Indochina allowed us to confirm five *Acromastigum* species and analyze the genetic structure of two of them. We assume that compiling a complete review of all species of this genus currently known from East Indochina should facilitate the understanding of this genus in Southeast Asia. To compile such a review was the purpose of the present account.

## Materials and methods

### Natural conditions in East Indochina

East Indochina, as identified here, covers Laos, Cambodia, and Vietnam and lies entirely south of the Tropic of Cancer. The relief is predominantly mountainous, although some areas consist of wide, flat, well-developed river valleys (especially prominent along the Mekong River). In the north, the orographic features are an extension of the Hengduan Mountains and continue as the 1100-km-long Annamite Range, ending with the Central Highlands (Lâm Viên Plateau) in Vietnam and the Cardamom and Elephant Mountains in Cambodia. The southern mountain ranges are dominated by the Am climate type (tropical monsoon climate), the main area is characterized by the Aw climate (tropical wet and dry or savanna climate), and the northern quarter is dominated by the Cwa climate (monsoon-influenced humid subtropical climate) [16,17]. East Indochina is the eastern part of the Indochinese floristic region, bordering the East Asian floristic region to the north and the Malesian floristic region to the south [18]. Landscape diversity, produced by the orographic features, climate variation and long genesis, has led to the formation of distinctive and taxonomically rich complexes, a general outline of which is provided by Averyanov & al. [19].

### The previous data on *Acromastigum* in East Indochina

Original data on the distribution of *Acromastigum* species in East Indochina come from several sources. *Acromastigum herzogii* in Vietnam was reported by Pócs & al. [20] and Pócs [21]. *Acromastigum echinatiforme* in Vietnam was mentioned by Pócs [22]. *Acromastigum echinatum* was mentioned by Shu et al. [23] with reference to Piippo [10], but the occurrence of this species in Vietnam was not mentioned in the cited Piippo paper. Therefore, the basis for this reference is unclear. *Acromastigum laevigatum* was mentioned by Jovet-Ast & Tixier [24]. Two species were previously reported from Cambodia. *Acromastigum inaequilaterum* was mentioned on the basis of the material studied ‘firsthand’ by Ingerpuu & al. [25] and Jovet-Ast [26]. *Acromastigum divaricatum* was reported by Bakalin & al. [27]. Data on the distribution of representatives of the genus in Laos are not yet known.

### Herbarium material

A total of 41 specimens of 6 species were studied, including 32 collected by us and 9 collected by other researchers. Most of the specimens are housed at VBGI and JNU, and three specimens are at NICH. For the specimens that our team collected, the geographic coordinates, community type, substrate, humidity, and lighting conditions were recorded during collection (the last two were identified visually, without the use of special equipment). Specimens collected in Cambodia were dried immediately after collection. Specimens from Vietnam were delivered alive to laboratories, where they underwent further examination, including the capture of ‘live’ photographs. Morphological studies were conducted at the Cryptogamic Biota Laboratory of the Botanical Garden of the Far Eastern Branch of the Russian Academy of Sciences (VBGI), Russia; the National Institute of Ecology, Republic of Korea; and the Laboratory of Plant Ecology and Remote Sensing of the Institute of Biology, Vietnam Academy of Science and Technology (HN), Vietnam. For most of the discussed taxa, we provide photographs taken using cameras associated with an Olympus CX43 (upright compound microscope) and an Olympus SZX16 (dissecting microscope) at VBGI, Nikon SMZ800N and Olympus BX43 microscopes at HN, and a Leica M205C dissecting microscope and an upright compound Leica DM2500 microscope at NIE. The specimens were examined using anatomical and morphological methods, and some were subjected to molecular genetic analysis (described below).

The field study in Xuan Son National Park was conducted in the frame of the VAST (Vietnam Academy of Science and Technology) project KHCBTĐ.02/21–23 (Project leader: Nguyen Van Sinh, Institute of Biology, VAST) and was conducted with permission from the Phu Tho People committee and the Management board of the Xuan Son National Park. The field study in Pù Mát National Park was conducted in the frame of the VAST project KHCBTĐ.02/21–23 (Project leader: Nguyen Van Sinh, Institute of Biology, VAST) and was conducted with permission from the Nghệ An People committee and the Management board of the Pù Mát National Park.

The field study in Vũ Quang National Park was conducted in the frame of the NAFOSTED (National Foundation for Science and Technology Development) project 106.03–2023.40 (Project leader: Nguyen Van Sinh, Institute of Biology, VAST) and was conducted with permission from the Hà Tĩnh People committee and the Management board of the Vũ Quang National Park.The field study in Bạch Mã National Park was conducted in the frame of the NAFOSTED project 106.03–2023.40 and was conducted with permission from the People committee of the Huế city and the Management board of the Bạch Mã National Park. The field study in Ngọc Linh Nature Reserve was conducted in the frame of the NAFOSTED project 106.03–2023.40 and was conducted with permission from the People committee of the Kon Tum Province (since July 01, 2025: Quảng Ngãi Province) and from the Management board of the Ngọc Linh Nature reserve. The field study in Bidoup Núi Bà National Park was conducted in the frame of the VAST project CBCLCA.14/25–27 (Project leader: Nguyen Van Sinh, Institute of Biology, VAST) and was conducted with permission from the Lâm Đồng People committee and the Management board of the Bidoup Núi Bà National Park.

### Sampling of taxa for molecular analysis

To compile the dataset for molecular phylogenetic analysis, we obtained a number of sequences available for the Lepidoziaceae family from potentially genetically related genera on the basis of the works published by He-Nygrén & al. [28], Heslewood and Brown [29], Cooper & al. [30–32], Katagiri & al. [33], and Rayos & al. [34] and by using BLAST (https://blast.ncbi.nlm.nih.gov/Blast.cgi accessed on 15.02.2026) search results. Available data were downloaded from the GenBank (National Center for Biotechnology Information (NCBI) 2026) database. Four species from our collection were included in the molecular analysis. Unfortunately, reliable sequences were obtained for only two of them. *Zoopsis argentea* (Hook. f. & Taylor) Gottsche, Lindenb. & Nees was selected as an outgroup for tree rooting following the general phylogeny of the family published by Cooper & al. [30,35]. Voucher specimen details, including GenBank accession numbers, are listed in S1 Appendix.

### DNA isolation, PCR amplification and DNA sequencing

DNA was extracted from dried liverwort tissues using the HiPure SF Plant DNA Kit (Magen, Huangpu District, Guangzhou, Guangdong, China) following the manufacturer’s protocol. Ampliﬁcation of the *trn*L–*trn*F, *trn*G–intron and *rbc*L loci was performed using an Encyclo Plus PCR Kit (Evrogen, Moscow, Russia) with the primers listed in Table 1.

**Table 1:** Primers used in polymerase chain reaction (PCR) and cycle sequencing.

Locus	Sequence (5′-3′)	Direction	Annealing Temperature (°C)	Reference
*trn*L–*trn*F cpDNA	CGAAATTGGTAGACGCTGCG	forward	62	[36]
*trn*L–trnF cpDNA	TGCCAGAAACCAGATTTGAAC	reverse	58	[36]
*trn*G-intron cpDNA	CGGGTAGCGGGAATCGAAC	forward	62	[37]
*trn*G-intron cpDNA	GCGGGTATAGTTTAGTGG	reverse	54	[38]
*rbc*L cpDNA	ATGTCACCACAAACAGAGACTAAAGC	forward	50	[39]
*rbc*L cpDNA	GCAGCAGCTAAMTCRGGACTCCA	reverse	50	[40]

The polymerase chain reaction was performed in a total volume of 20 µl, including 1 µl of template DNA, 0.4 µl of Encyclo polymerase, 5 µl of Encyclo buffer, 0.4 µl of dNTP mixture (included in the Encyclo Plus PCR Kit), 13.4 µl (for *trn*L–F, *trn*G–intron)/12.4 µl (for *rbc*L) of double-distilled water (Evrogen, Moscow, Russia), 1 µl of dimethylsulfoxide (for *rbc*L), and 0.4 µl of each primer (forward and reverse, at a concentration of 5 pmol/µl). Polymerase chain reactions were performed by using the following amplification protocol:

*Initial denaturation* at 95°C for 3 min;
*Denaturation* at 94°C for 30 sec (*trn*L–F, *trn*G–intron) or 40 sec (*rbc*L);
*Annealing* at 50°C (*rbc*L), 56 °C (*trn*G–intron) or 58°C (*trn*L–F) for 20 sec (*trn*L–F), 30 sec (*trn*G–intron) or 90 sec (*rbc*L);
*Elongation* at 72°C for 30 sec (*trn*L–F, *trn*G–intron) or 2 min (*rbc*L);
*Final elongation* at 72°C for 5 min.

The “*Denaturation,*” “*Annealing*” and “*Elongation*” steps were repeated for 35–37 cycles.

Ampliﬁed fragments were visualized after electrophoresis on 1.2% agarose TAE (Tris-acetate-EDTA) gels by EthBr staining and puriﬁed using the HiPure Gel DNA Mini Kit (Magen, Huangpu District, Guangzhou, Guangdong, China). The DNA was sequenced using the ABI PRISM® BigDye™ Terminator Cycle Sequencing Ready Reaction Kit (Applied Biosystems, Foster City, CA, USA), and the reaction products were further analyzed on an automatic sequencer (3730 DNA Analyzer, Applied Biosystems, USA) at the Genome Center (Engelhardt Institute of Molecular Biology, Russian Academy of Sciences, Moscow, Russia) as per the standard protocol.

### Phylogenetic analysis

Datasets were produced for the *trn*L–F, *trn*G and *rbc*L loci, and preliminary trees constructed for the *trn*L–F and *rbc*L loci were congruent; accordingly, we combined them into one tree. The general topology obtained using the *trn*G locus was also congruent with that for the *trn*L–F and *rbc*L loci, but we could not construct a consensus tree that included that locus because the original data were from specimens different from those for the *trn*L–F and *rbc*L loci. Owing to this constraint, we constructed a separate tree for the *trn*G locus. All sequences were aligned using MAFFT [41] with standard settings and then edited manually in BioEdit ver. 7.2.5 [42]. All positions of the final alignments were included in the phylogenetic analyses. All missing positions in the alignments at the beginnings and ends of regions and gaps were treated as missing data.

Phylogenies were reconstructed under two criteria: maximum likelihood (ML) with IQ-tree ver. 2.2.2.6 [43] and Bayesian inference (BI) with MrBayes ver. 3.2.7 [44]. For the ML analysis with 1000 bootstrap replicates, the best-fitting evolutionary model of nucleotide substitutions according to the BIC value was K3Pu + F + I + R2 for *trn*L‒F + *rbc*L and K3Pu + F + G4 for *trn*G, as determined by ModelFinder (model selection method implemented in IQ-tree) [45]. Bootstrap support (BS) percentage values were calculated.

BI trees were constructed by running two parallel analyses using the general time-reversible (GTR) model space (nst = mixed). For all datasets, the analysis consisted of four Markov chains run for 5,000,000 generations, and trees were sampled every 500th generation. The first 2500 trees in each run were discarded as burn-in; thereafter, 15000 trees were sampled from both runs. Bayesian posterior probabilities (PPs) were calculated from the trees sampled after burn-in. The average standard deviation of split frequencies between the two runs was 0.0013 for *trn*G and 0.0041 for *trn*L‒F + *rbc*L.

Infraspecific and interspecific variation were quantified as the average pairwise *p*-distances calculated in Mega XII [46] using the pairwise deletion option for counting gaps, the proportion of nucleotide sites that are different (d: Transitions + Transversions), and 1000 bootstrap replicates.

Considering the limitations of classical cladistics (phylogenetic trees) in visualizing relationships at the molecular level for closely related species, we carried out this task by applying a method called split networks, implemented in SplitsTree v.4.14.2 [47] software.

## Results

### Molecular phylogeny

**Fig 1:**
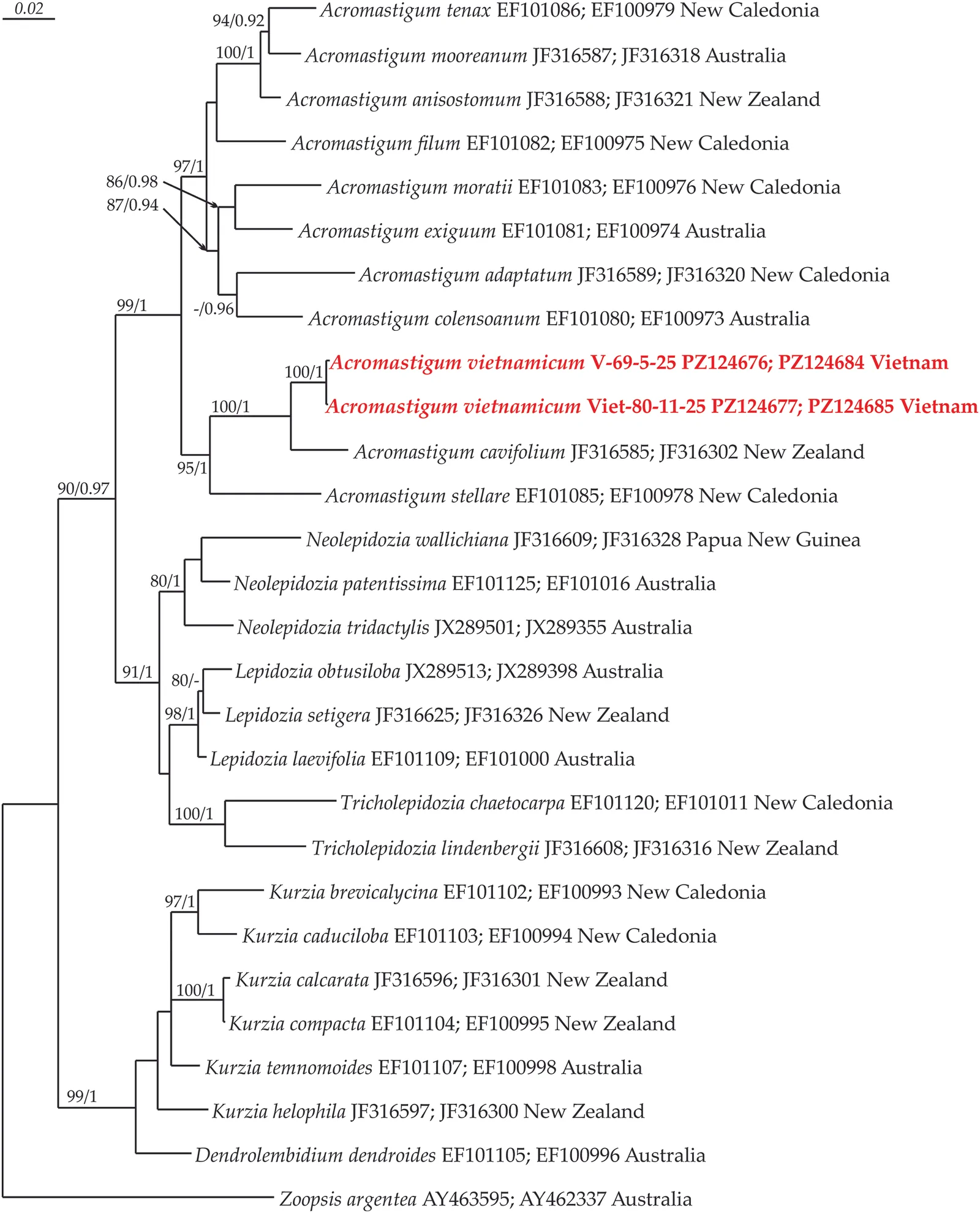
Phylogram obtained from a Bayesian analysis for the Lepidoziaceae family from potentially genetically related genera based on *trn*L‒F plus *rbc*L loci. Newly obtained sequences are marked in red plus bold. Bootstrap support values (BS) > 80% in ML analysis and Bayesian posterior probabilities (PP) > 0.80 are indicated. Scale bar denotes the number of nucleotide substitutions per site.

Ten sequences (four of *trn*L‒F, four of *trn*G and two of *rbc*L) were newly produced and deposited into GenBank. Since we provide the consensus *trn*L‒F + *rbc*L tree but we obtained *rbc*L sequences for only two specimens, we used only two *trn*L‒F sequences in our phylogenetic tree. The *trn*L‒F + *rbc*L phylogeny is shown in Fig 1. The alignment of 28 sequences consisted of 1124 positions, of which 227 were parsimony-informative sides, 141 were singleton sites and 756 were constant sites. The base frequencies across all the sites were as follows: A, 0.317; C, 0.168; G, 0.195; T, 0.321. The ML criterion resulted in a consensus tree with a log likelihood of −5503.229. The arithmetic means of the log likelihoods from the Bayesian analysis of each sampling run were −5520.42 and −5520.92.

**Fig 2:**
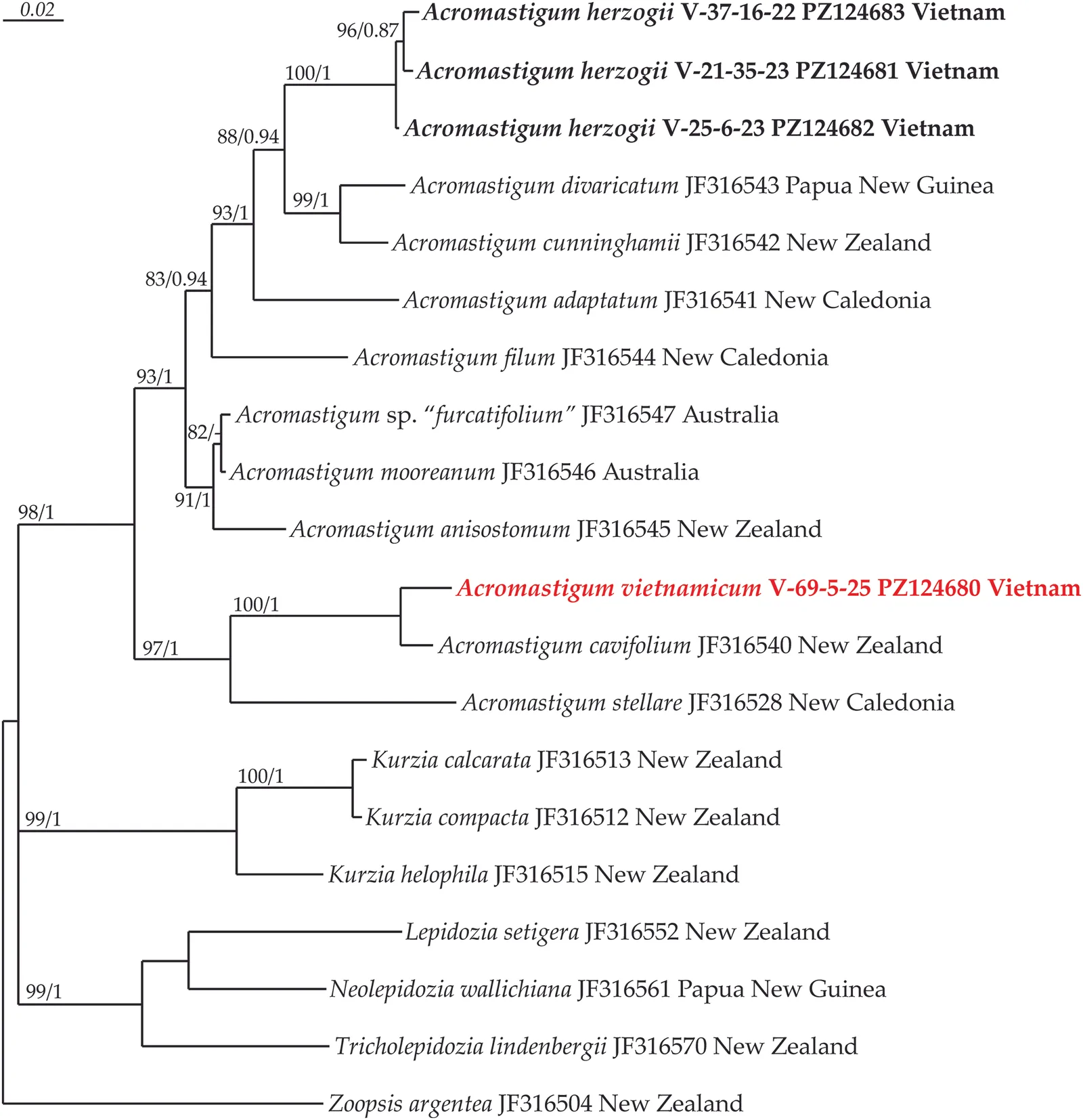
Phylogram obtained from a Bayesian analysis for the Lepidoziaceae family from potentially genetically related genera based on *trn*G. Newly obtained sequences are marked in bold and in red plus bold. Bootstrap support values (BS) > 80% in ML analysis and Bayesian posterior probabilities (PP) > 0.80 are indicated. Scale bar denotes the number of nucleotide substitutions per site.

The *trn*G phylogeny is shown in Fig 2. The alignment of 20 sequences consisted of 597 positions, of which 158 were parsimony informative, 101 were singletons and 338 were constant sites. The base frequencies across all the sites were as follows: A, 0.358; C, 0.158; G, 0.125; T, 0.358. The ML criterion resulted in a consensus tree with a log likelihood of −3006.677. The arithmetic means of the log likelihoods from the Bayesian analysis of each sampling run were −3009.55 and −3009.67.

The topologies of the trees we constructed yielded similar results: the studied accession of the potentially new species fell into a clade with *Acromastigum stellare*, with BS/PP support values of 95/1 (*trn*L‒F + *rbc*L tree) and 97/1 (*trn*G tree), and *Acromastigum cavifolium*, with BS/PP support values of 100/1 on both trees. The genetic distances (Tables 2–4) between the potentially new species and *A. cavifolium* were found to be 2.89% for *trn*L‒F, 2.73% for *rbc*L and 2.44% for *trnG*. These distances roughly correspond to the distances between the phylogenetically closest species in other clades. Thus, in the presence of distinguishing morphological features, there are no contraindications for considering the new taxon at the species level. The newly obtained sequences for *Acromastigum herzogii* (*trn*G only) are placed in the sister branch with the clade *A. divaricatum* + *A. cunninghamii* with 88/0.94 support values. The phylogenetic tree (Fig 2) shows the undoubted placement of *A. herzogii* together with taxa belonging to subg. *Inaequilatera* (but not to subg. *Acromastigum* as in Söderström & al. [1].

**Table 2:** Interspecific *p*-distances for the family *Acromastigum* based on *trn*L–F locus currently available nucleotide sequence data. The number of base differences per site from averaging over all sequence pairs between each group is shown. Infraspecific *p*-distance within the *Acromastigum vietnamicum* group was 0%, *A. herzogii* ‒ 0,63%, other groups ‒ n/c (not calculated due to the presence of a single specimen). The distances are sorted in ascending order relative to *A. vietnamicum*. Newly obtained sequences are bolded.

№	Taxa	Interspecific *p*-distances, *trn*L–*trn*F, %										
		1	2	3	4	5	6	7	8	9	10	11
1	***A. vietnamicum***											
2	*A. cavifolium*	**2.89**										
3	*A. stellare*	**9.33**	10.02									
4	*A. mooreanum*	**9.8**	11.53	11.28								
5	*A. sp “furcatifolium”*	**9.8**	11.53	11.28	0							
6	*A. tenax*	**9.8**	11.31	10.4	3.23	3.23						
7	*A. filum*	**9.82**	11.56	10.86	7.73	7.73	7.06					
8	*A. exiguum*	**10.68**	10.43	10.16	9.46	9.46	9.23	8.35				
9	*A. colensoanum*	**11.97**	12.39	11.23	9.89	9.89	9.67	7.93	8.07			
10	*A. moratii*	**12.11**	12.72	11.36	11.53	11.53	10.86	10.89	7.48	9.07		
11	*A. adaptatum*	**12.72**	12.95	15.11	11.97	11.97	11.53	11.11	11.09	10.82	13.84	
12	***A. herzogii***	**13.27**	13.83	13.19	12.85	12.85	12.7	11.67	11.21	7.88	12.61	12.01

**Table 3:** Interspecific *p*-distances for the family *Acromastigum* based on *rbc*L locus currently available nucleotide sequence data. The number of base differences per site from averaging over all sequence pairs between each group is shown. Infraspecific *p*-distance within the *Acromastigum vietnamicum* group was 0,09%, *A. mooreanum* ‒ 0%, other groups ‒ n/c (not calculated due to the presence of a single specimen). The distances are sorted in ascending order relative to *A. vietnamicum*. Newly obtained sequences are bolded.

№	Taxa	Interspecific *p*-distances, *rbc*L, %									
		1	2	3	4	5	6	7	8	9	10
1	***A. vietnamicum***										
2	*A. cavifolium*	**2.73**									
3	*A. filum*	**4.9**	5.3								
4	*A. colensoanum*	**5.08**	5.3	3.13							
5	*A. stellare*	**5.51**	5.64	5.21	4.95						
6	*A. exiguum*	**5.51**	5.47	3.47	3.47	5.38					
7	*A. sp “furcatifolium”*	**5.86**	6.25	3.21	3.82	5.56	3.99				
8	*A. mooreanum*	**5.86**	6.25	3.21	3.82	5.56	3.99	0			
9	*A. moratii*	**5.95**	6.17	3.48	3.82	5.99	3.74	4.52	4.52		
10	*A. tenax*	**6.03**	6.77	3.65	4.25	5.82	4.34	2.43	2.43	4.78	
11	*A. adaptatum*	**6.55**	6.68	4.08	3.91	6.51	3.91	4.77	4.78	3.82	4.77

**Table 4:** Interspecific *p*-distances for the family *Acromastigum* based on *trn*G locus currently available nucleotide sequence data. The number of base differences per site from averaging over all sequence pairs between each group is shown. Infraspecific *p*-distance within the *Acromastigum herzogii* ‒ 0,49%, other groups ‒ n/c (not calculated due to the presence of a single specimen). The distances are sorted in ascending order relative to *A. vietnamicum*. Newly obtained sequences are bolded.

№	Taxa	Interspecific *p*-distances, trnG, %									
		1	2	3	4	5	6	7	8	9	10
1	***A. vietnamicum***										
2	*A. cavifolium*	**2.73**									
3	*A. mooreanum*	**4.9**	5.3								
4	*A. sp “furcatifolium”*	**5.08**	5.3	3.13							
5	*A. anisostomum*	**5.51**	5.64	5.21	4.95						
6	*A. stellare*	**5.51**	5.47	3.47	3.47	5.38					
7	*A. cunninghamii*	**5.86**	6.25	3.21	3.82	5.56	3.99				
8	*A. filum*	**5.86**	6.25	3.21	3.82	5.56	3.99	0			
9	*A. divaricatum*	**5.95**	6.17	3.48	3.82	5.99	3.74	4.52	4.52		
10	*A. herzogii*	**6.03**	6.77	3.65	4.25	5.82	4.34	2.43	2.43	4.78	
11	*A. adaptatum*	**6.55**	6.68	4.08	3.91	6.51	3.91	4.77	4.78	3.82	4.77

**Fig 3:**
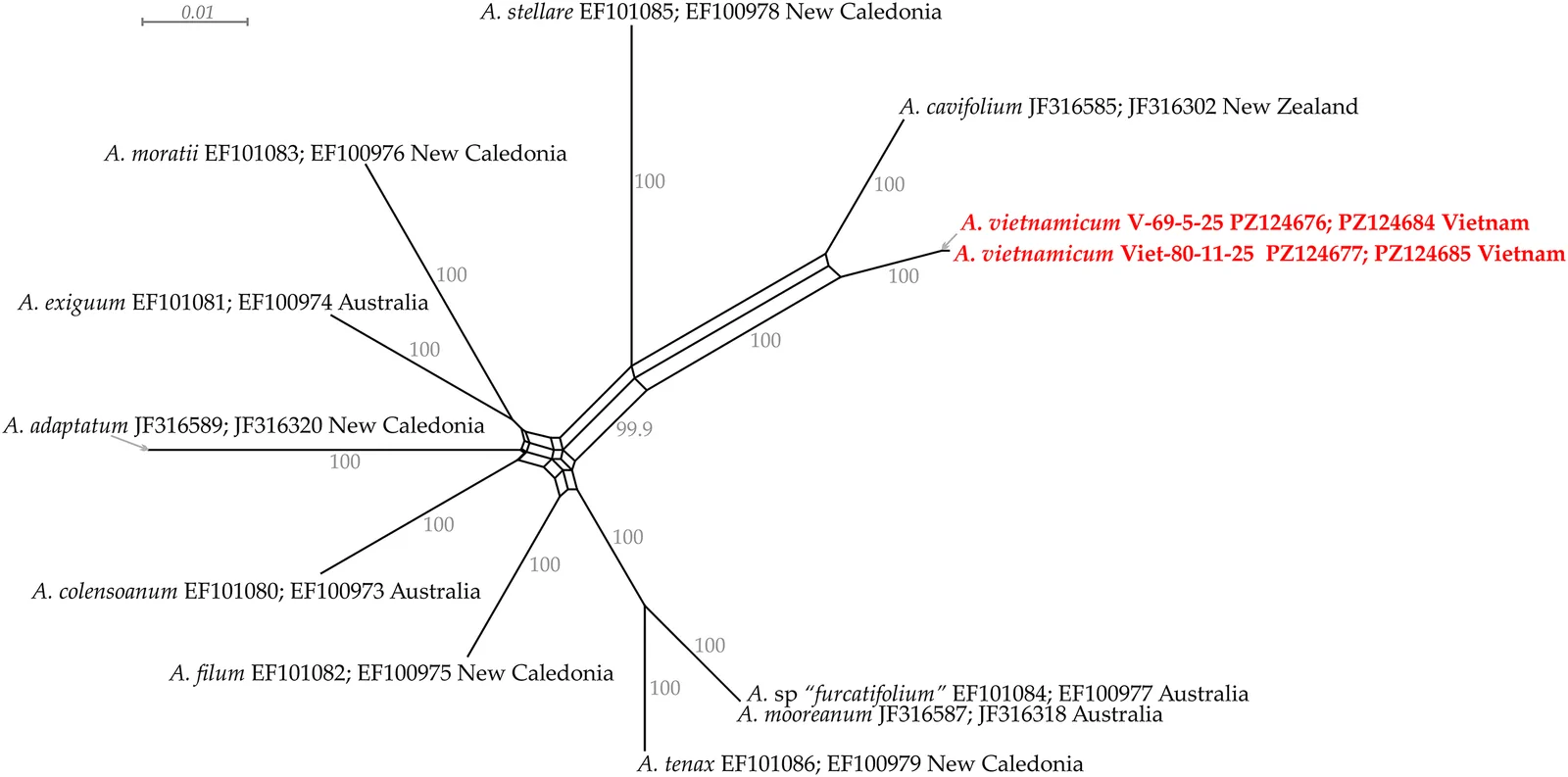
NeighborNet split network for *Acromastigum* specimens based on the *trn*L–*trn*F plus *rbc*L dataset. Newly obtained sequences are marked in red plus bold. Bootstrap support values of 95 or higher are indicated.

A SplitsTree analysis (Fig 3) evaluating horizontal haplotype similarity revealed a somewhat different pattern in the potentially new species. This species is also most closely related to *Acromastigum cavifolium*. However, *A. stellare* is equidistant from both the *A. cavifolium* + potentially new species pair and from all other *Acromastigum* species belonging to subg. *Inaequilatera*. Calculating *p*-distances (Tables 2–4) showed that *A. stellare* is equidistant from both groups, except for Table 4 (*trn*G-based), where shorter distances, on average, separate it from representatives of subg. *Inaequilatera*. Therefore, it seems more natural to place *A. stellare* into subg. *Inaequilatera*.

### Morphology

Among the six species reported for East Indochina, we examined specimens of five species from our own materials. Another reported species (*Acromastigum echinatiforme*) was not found in the materials available to our team. Several specimens from other areas were also examined. The specimen origins were as follows: *Acromastigum laevigatum*, 1 specimen from Cambodia; *A. echinatum*, 8 specimens (Cambodia, 6; Indonesia, 2); *A. inaequilaterum*, 11 specimens (Cambodia, 8; Indonesia, 3); *A. herzogii*, 14 specimens (Vietnam, 11; Thailand, 3). The new taxon described below as *Acromastigum vietnamicum* had 5 specimens from Vietnam. In addition, we examined a specimen that apparently represents an isotype of *Acromastigum moratii* from New Caledonia.

Morphological comparison of the specimens used as the basis for the report of *Acromastigum curtilobum* A. Evans from Thailand [14] (labeled in the NICH as “*Acromastigum angustilobum* N. Kitag.”, which seems to be a *nomen herbariorum*) showed that they should be referred to *A. herzogii*. Another specimen from Thailand, housed in NICH under the name *A. divaricatum*, which apparently has never been mentioned in publications, belongs to *A. herzogii* as well. Specimen C9299 cited by Bakalin et al. [27] under *A. divaricatum* actually belongs to *A. inaequilaterum*. We found no other reports referring to *A. curtilobum* or *A. divaricatum* in East. Indochina. We did not study *A. echinatiforme* specimens from East Indochina. From the study of available materials, morphological descriptions were compiled, and photographs were obtained (in the vast majority of cases, they are based on our own material).

The observed distribution of rhizoids deserves special attention. Typically, the literature states that rhizoids are scarce, without specifying their place of origin; states that they arise from the bases of underleaves or reduced leaves and underleaves in ventral flagella [4,11]; or, as in Evans [7], simply states that they are formed from cells of reduced leaves of flagella branches. We, however, noted that rhizoids develop from the bases of scale-like leaves and underleaves only in *Acromastigum vietnamicum* (subg. *Acromastigum*) described here, whereas in all the other species discussed here (subg. *Inaequilatera*), they arise strictly from the uppermost cells of scale-like leaves and underleaves.

### Taxonomic treatment

***Acromastigum echinatiforme*** (De Not.) A.Evans, Ann. Bryol., Suppl. 3: 64, 1934

≡ *Mastigobryum echinatiforme* De Not. Epat. Borneo 38. 1874

*Distribution.* – The species area covers Malesia, Melanesia, and Australasia [4,7,48]. The locality in Vietnam is at the extreme northwestern end of the species range and is the only report of the taxon in the Southeast Asian mainland.

*Comment.* – The species was reported for Vietnam by Pócs [22] from Hà Giang Province (environs of Phó Bảng, mossy forest, on tree bark, 1800 m a.s.l. No. ЗЗ/g.). The species is characterized by obliquely inserted leaves (the insertion line is without a sharp bend), with slightly unequal (in length and width) lobes composed of cells of approximately equal size, as well as underleaves whose length is slightly shorter than their width, and the lobes are 2–6 cells wide. Materials on this species were not available to us. Description and illustrations are provided by Evans [7], Brown & Renner [4] and Grolle [48]. The latter paper indicates that plants referred to this species from Australia differ quite significantly in size from the type plants from Malesia. Despite its name (which suggests a relationship with *A. echinatum*), this species may be confused with *A. divaricatum*. However, *A. echinatiforme* differs from *A. divaricatum* in the following ways: 1) the stem wall cells acquire secondary (brownish) pigmentation; 2) in the stem cross section, the cells of *A. echinatiforme* are so strongly thickened that, although formally the outer cells are larger, the lumens of the inner and outer cells are approximately the same diameter; 3) underleaves 1.5–2.0 times wider than the stem in *A. echinatiforme*, whereas narrower than the stem or up to 1.4 of stem in *A. divaricatum*.

***Acromastigum echinatum*** (Gottsche) A. Evans, Ann. Bryol., Suppl. 3: 147, 1934

≡ *Mastigobryum echinatum* Gottsche Syn. Hepat. 218. 1845

**Fig 4:**
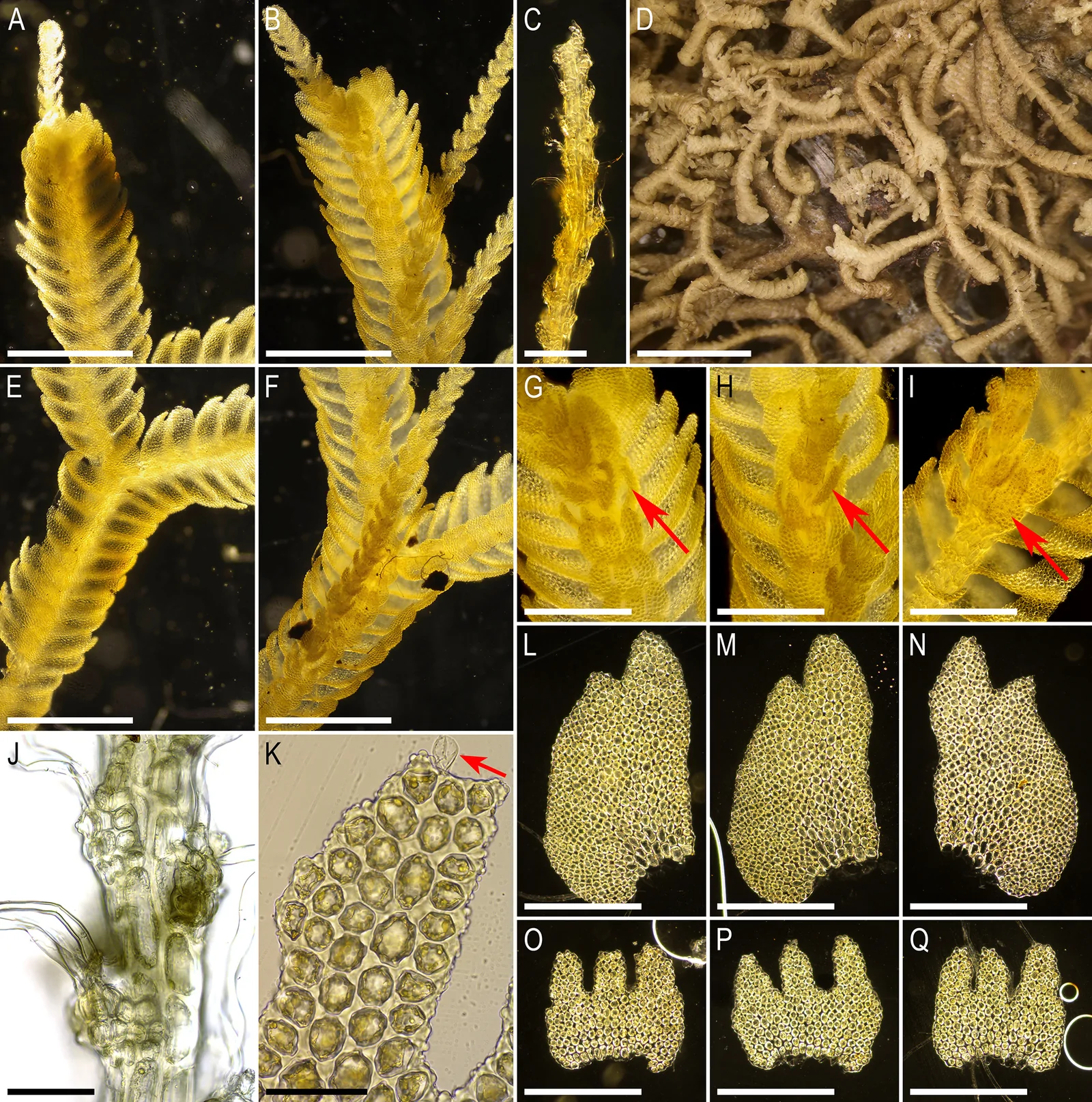
*Acromastigum echinatum* (Gottsche) A. Evans. (A) Upper part of the shoot with ventral flagella, dorsal view. (B) Upper part of the shoot with ventral flagella, ventral view. (C) Ventral flagella. (D) Part of the mat in dry condition. (E) Middle part of the shoot with *Frullania*-type branching, dorsal view. (F) Middle part of the shoot with ventral flagella, ventral view. (G–I) Fragments of shoot middle parts with 1-lobed underleaves at the flagella bases indicated by red arrows. (J) Fragment of ventral flagella with rhizoids, originating from the terminal cells of scale-like leaves. (K) Underleaf lobe with slime papilla indicated by red arrow. (L–N) Leaves. (O–Q) Underleaves. A–C, E–I, L–Q are photographed with dark field option. Scales: 4 mm for D; 1 mm for A, B, E, F; 500 µm for C, G–I; 300 µm for L–Q; 100 µm for J; 50 µm for K. A–J, L–Q from *Cam-87-15-11* (VBGI); K from *Cam-87-14-11* (VBGI).

**Fig 5:**
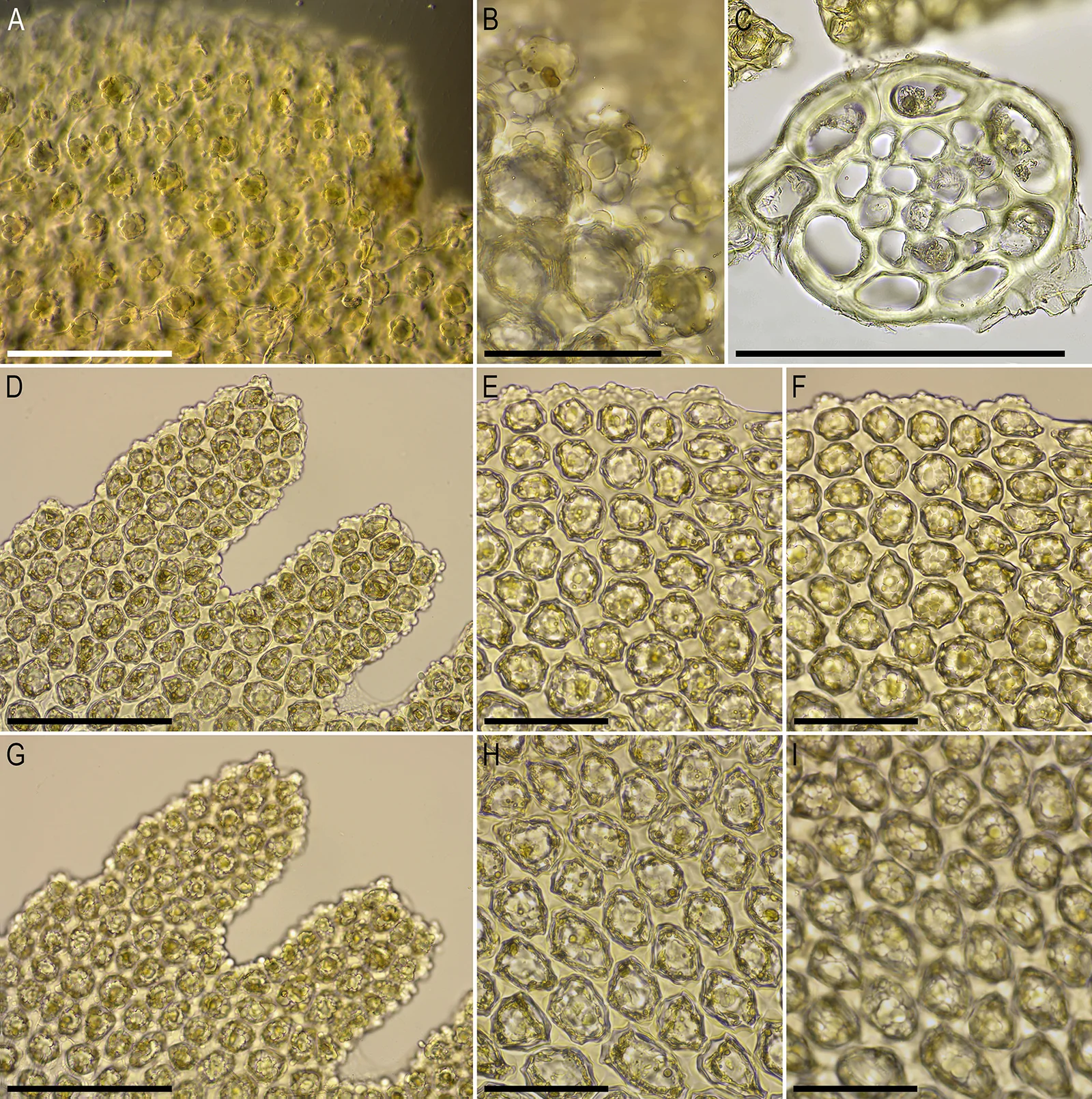
*Acromastigum echinatum* (Gottsche) A. Evans. (A) Clusters of papillae over apical parts of mamillosely projecting cells in upper part of the leaf. (B) Mamillosely projecting cells covered by papillae. (C) Stem cross-section. (D) Cells of underleaf lobes. (E) Cells of leaf margin. (F) Cells of leaf margin with papillae. (G) Cells of underleaf lobes with clusters of papillae over apical parts of mamillosely projecting cells. (H) Midleaf cells. (I) Midleaf cells with papilla clusters. A is photographed with dark field option. Scales: 200 µm for C; 100 µm for A, D, G; 50 µm for B, E, F, H, I. A from *Cam-87-11-11* (VBGI); B from *Cam-87-8-11* (VBGI); C–I from *Cam-87-15-11* (VBGI).

*=Jungermannia inaequilatera* var. *minor* Lehm. & Lindenb. Nov. Stirp. Pug. 6: 56. 1834 (Figs 4 and 5)

*Description.* – Plants prostrate, loosely attached to the substratum, freely branched and forming compact mat, pale green when fresh and yellowish brownish when dry in herbarium after 10 years of keeping, fragile and with leaves distinctly turned ventrally when dry (always loose the ventral flagella), 0.8–1.0 mm wide and 8–20 mm long (older parts decaying and decomposing), branching of the main stem of *Frullania*-type (modified leaf ovate in general outline, unlobed, acute at apex), ventral branching resulting in geotropic positive flagella of *Acromastigum*-type, instead of 1–2 of 3 underleaf lobes (therefore 1–2 lobes remained). Stem cross section slightly transversely ellipsoidal, ca. 150 × 200 µm, with 7–8 outer cells 25–35 × 35–50 µm, with thickened walls, outer wall noticeable thick, trigones large, concave, outer cuticle smooth; inner cells 13–25 µm in diameter, with slightly thickened walls and large, triangle to slightly convex trigones. Rhizoids scattered in the terminal portions of flagella, colorless, flexuous, 150–300 µm long, originating from the terminal cells of scale-like leaves or underleaves (indistinguishable), separated, obliquely spreading. Leaves imbricate, covering ca. 1/2 of the above situated leaf in its basal part, obliquely inserted and oriented, suberect spreading, distinctly turned to the ventral side, convex, when flattened obliquely ovate, unequally bilobed, 500–600 × 350–400 µm in well-developed shoots, divided by V- to U-shaped sinus descending to 1/5 of the leaf length (actually difficult to measure, because lobes are unequal in the length) into two strongly unequal lobes with rounded apices, dorsal lobe distinctly smaller. Midleaf cells 25–40 × (20–)22–27 µm, thin-walled, distinctly papillose (longer cells) to verrucose (subisodiametric cells), verrucae distributes above the cell lumen, while the papillae may stretch across the cell walls; becoming distinctly smaller to the margins, near dorsal margin subisodiametric, 15–20(–25) µm in diameter, with thin walls, but external wall strongly thickened, strongly verrucose with verrucae restricted to the central part of the cell lumen, additionally the cells are mamillosely projecting above the surface. Underleaves transversely ellipsoidal loosely appressed to the stem commonly covering the lower part of the above situated underleaf, divided into 3 subequal lobes by the V- to U-shaped sinus descending to 1/2 of the underleaf length, lobe apices rather rounded to rectangular, with early deciduous slime papilla in the middle part of lobe apex, when flattened in the slide 270–300 × 300–350 µm, surface prominently verrucose (verrucae size 2–5 µm in diameter) above the cell lumen, but not above the cell walls.

*Specimens examined.* – CAMBODIA: Koh Kong Province, Trapeang Rung, 11.59694°N, 103.22556°E, 380 m a.s.l., wet broadleaved evergreen forest at the edge of grassland, decaying wood, 25 Dec 2011, *V.A. Bakalin Cam-87-8-11, Cam-87-10-11, Cam-87-14-11, Cam-87-15-11* (VBGI); MALAYSIA: Johore Endau-Rompin, wet ground in swamp, 22 Apr 1986, *Kiew Bong Heang 48* (NICH 424265!); INDONESIA: Sumatra, Padang bij Pementas, 28 Jul 1953, *Kostermans 19a* (NICH 216293!).

*Ecology.* – Our specimens were collected on a decaying decorticated fallen tree trunk in wet evergreen forest at the edge of the grassland at quite low elevation (380 m a.s.l.). The data on ecology provided by Piippo [10: page 30] are rather scarce and indicate only ‘primary forest’.

*Distribution.* – The species distribution extends from Indochina (Cambodia only) through Malesia (including Singapore on the Malay Peninsula) and reaches Melanesia [7,48].

**Fig 6:**
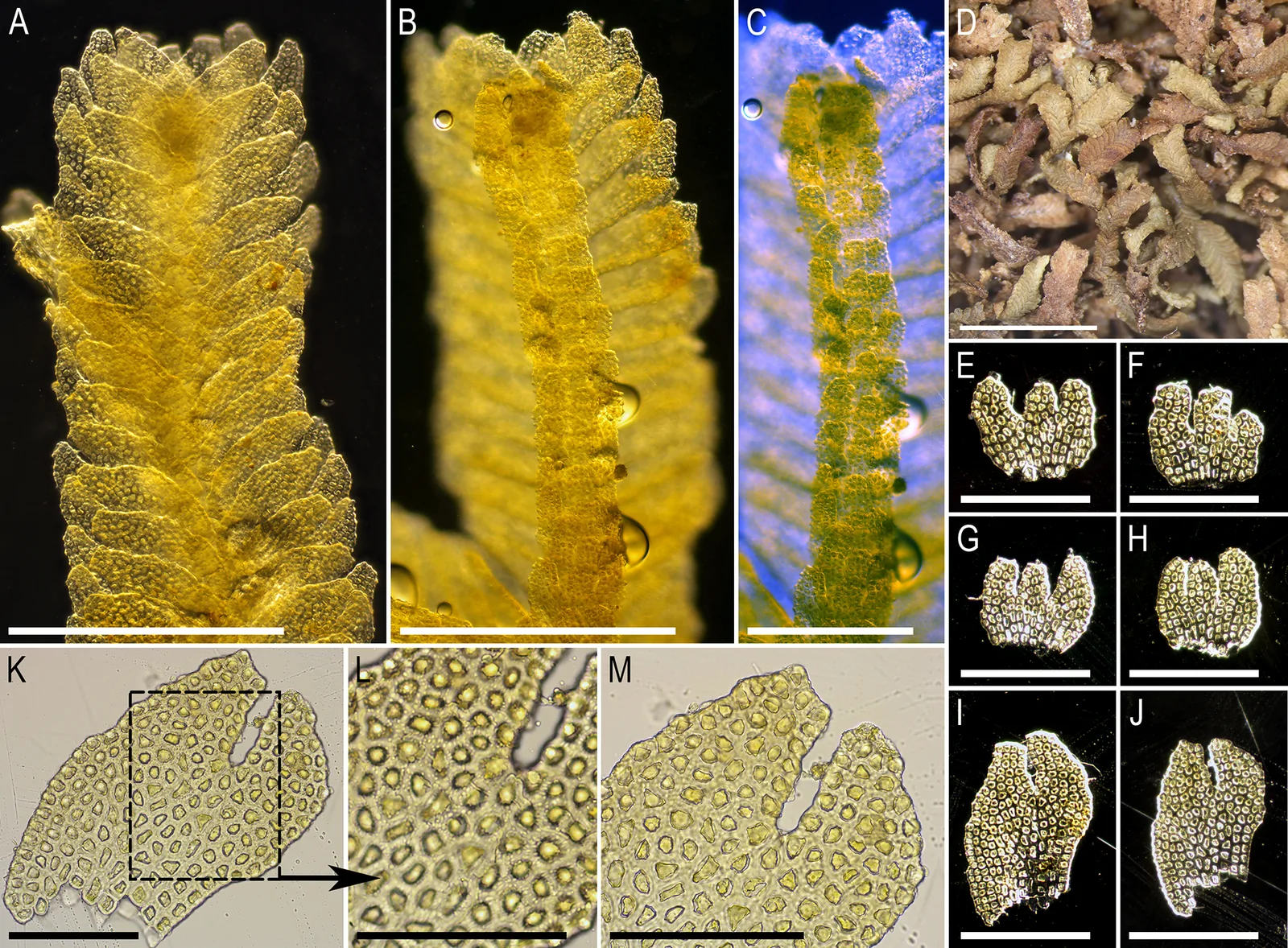
*Acromastigum moratii* N. Kitag. (A) Shoot with ventral flagella, dorsal view. (B) Shoot, ventral view. (C) Underleaves in an upper part of a shoot, ventral view (original colors have been changed). (D) Part of a mat in dry condition. (E–H) Underleaves. (I, J) Leaves. (K) Leaf cells. (L) Enlarged portion of K with mamillosely projecting cells covered by papillae. (I) Cells of upper half of a leaf. A–C, E–J are photographed with dark field option. Scales: 2 mm for D; 500 µm for A, B; 300 µm for C; 200 µm for E–J; 100 µm for K–M. All from isotype from exsiccate no. 184, published as *A. echinatum, Kitagawa BE186* (VBGI).

*Comment.* – This species belongs to a group of morphologically similar taxa with somewhat falcate leaf lobes that includes, in addition to *Acromastigum echinatum*, *A. inaequilaterum* (Lehm. et Lindenb.) A. Evans and *A. moratii* N. Kitag. (Fig 6) [12,48]. The morphological similarity of *A. echinatum* and *A. inaequilaterum* is discussed by Evans [7] as well. The World Liverwort Checklist regards *A. echinatum* as a species with doubtful status (marked with one asterisk), stating ([1] footnote 105) that “*Acromastigum echinatum* may be conspecific with *Acromastigum inaequilaterum* since all specimens identified by Piippo & al. [49] belong there”. However, it remains unclear to us how the incorrect identifications in the aforementioned work by Piippo can indicate the potential synonymy of the two names. The distinguishing features, as they are provided by Evans [7], Grolle [48] and Kitagawa [12], for *A. echinatum* and the other two taxa seem quite definite and can be summarized as follows: 1) *A. inaequilaterum* and *A. moratii* have smaller cells (in *A. moratii* leaf lobes, varying in the range of approximately 12–26 µm; in the middle 18–24 × 12–16 µm; in *A. inaequilaterum*, mostly 20–30 × 20–25 µm in the midleaf and diminishing to 15 µm to the margins). 2) *A. inaequilaterum* and *A. moratii* possess strongly and evenly thickened leaf cell walls such that the trigones are hardly observable, and if present, concave (versus cell walls are thin and trigones are prominent and commonly convex in *A. echinatum*). 3) *A. inaequilaterum* commonly possesses true teeth along the leaf margin, not the *Allorgella*-type denticulations due to protruding radial cell walls or mammillose protrusions of cell walls as in *A. moratii* and *A. echinatum*. Additionally, when in a dry state in the herbarium, *A. inaequilaterum* appears darker in color (to a pale brownish) and has less ventrally curved to ventral side leaves than in *A. echinatum*. All three species typically possess mammillose protrusions of cell walls over the leaf surface, terminated by a clusters of verrucae. The latter feature, however, seems not universal. Surface smoothing sometimes occurs in *A. inaequilaterum*. The New Caledonian *A. moratii* is supposedly closely similar to *A. echinatum*, as might be because the descriptions. However, it clearly differs even at first glance. *Acromastigum moratii* (isotype from exsiccate no. 184, published as *A. echinatum*, 980 m alt., on decaying log, N. Kitagawa, 29 July 1982) is clearly distinguished by its prostrate, feather-shaped plants with leaves not reflexed ventrally and a brownish coloration with a distinct admixture of reddish pigmentation (not present in *A. inaequilaterum* and *A. echinatum*) and generally much smaller plant size than the other two species. The corresponding photographs of the latter are provided here.

Unfortunately, despite repeated attempts to obtain sequences for *A. echinatum* specimens listed above for comparison with the sequences available in GenBank for *A. moratii*, we were unsuccessful.

**Fig 7:**
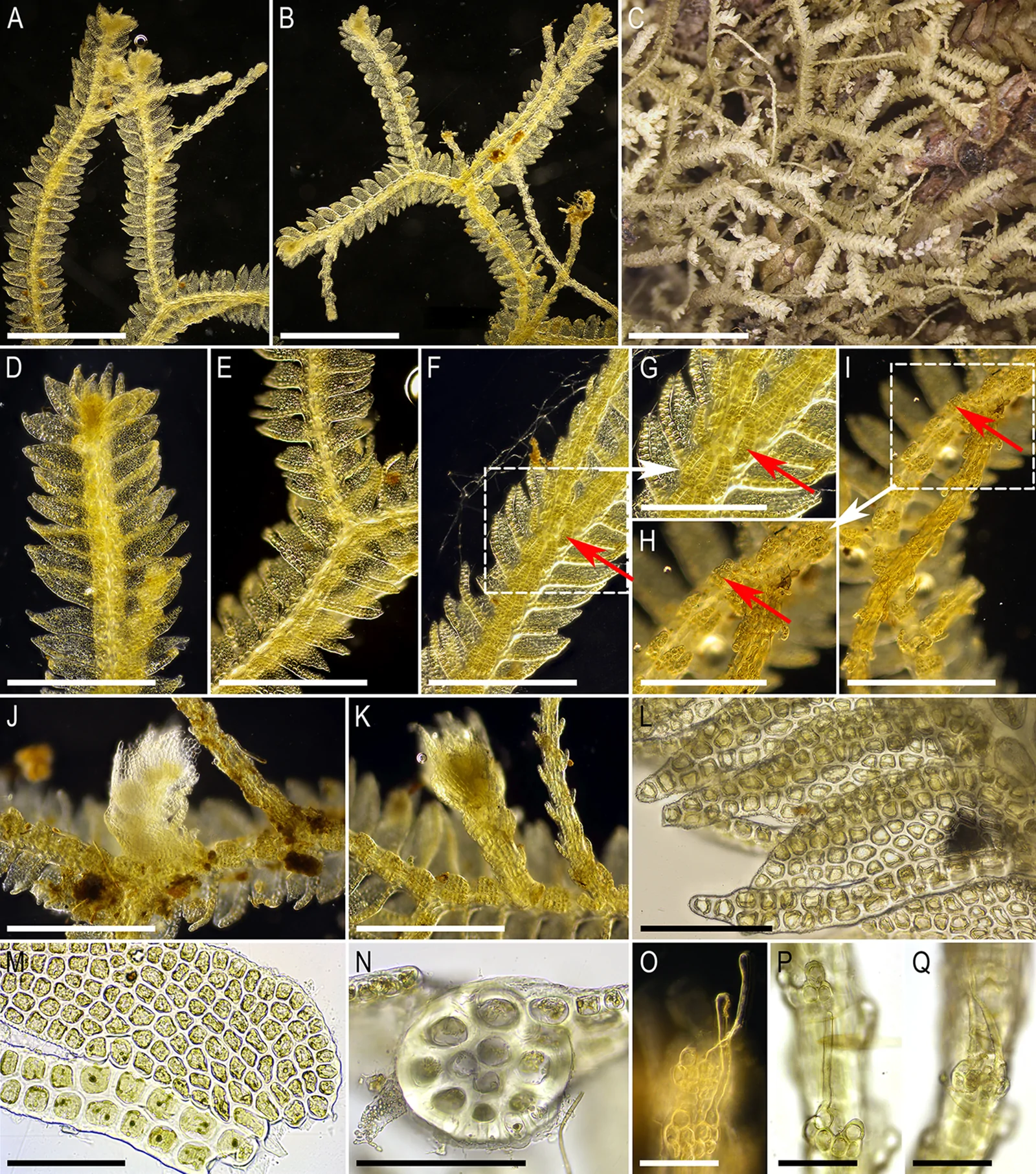
*Acromastigum herzogii* Grolle. (A) Shoots with ventral flagella, dorsal view. (B) Shoot with ventral flagella, ventral view. (C) Part of the mat in dry condition. (D, E) Fragments of shoots, dorsal view. (F, I) Fragments of shoot middle parts with 1-lobed underleaves at the flagella bases indicated by red arrows, ventral view. (G) Enlarged portion of F. (H) Enlarged portion of I. (J) Androecia on short ventral branch. (K) Archegonia on short ventral branch. (L) Leaves inserted to the stem, fragment, dorsal view. (M) The lower part of the leaf, cells in the ventral and dorsal lobes, strongly differ in size. (N) Stem cross-section. (O–Q) Rhizoids produced from apical cells of the scale-like leaves and underleaves on ventral flagella. A, B, D–K, O are photographed with dark field option. Scales: 3 mm for C; 1 mm for A, B; 500 µm for D–F, I, J, K; 300 µm for G, H; 100 µm for L, N–Q; 50 µm for M. A–C, E, M–Q from *V-25-5-23* (VBGI, dupl. in HN); D, L from *Tagawa & Kitagawa T1673* (NICH 277677, dupl. in VBGI); F, G from *V-36-25-22* (VBGI, dupl. in HN); H, I from *Touw 12173* (NICH 297393, dupl. in VBGI); J, K from *V-25-6-23* (VBGI, dupl. in HN).

**Fig 8:**
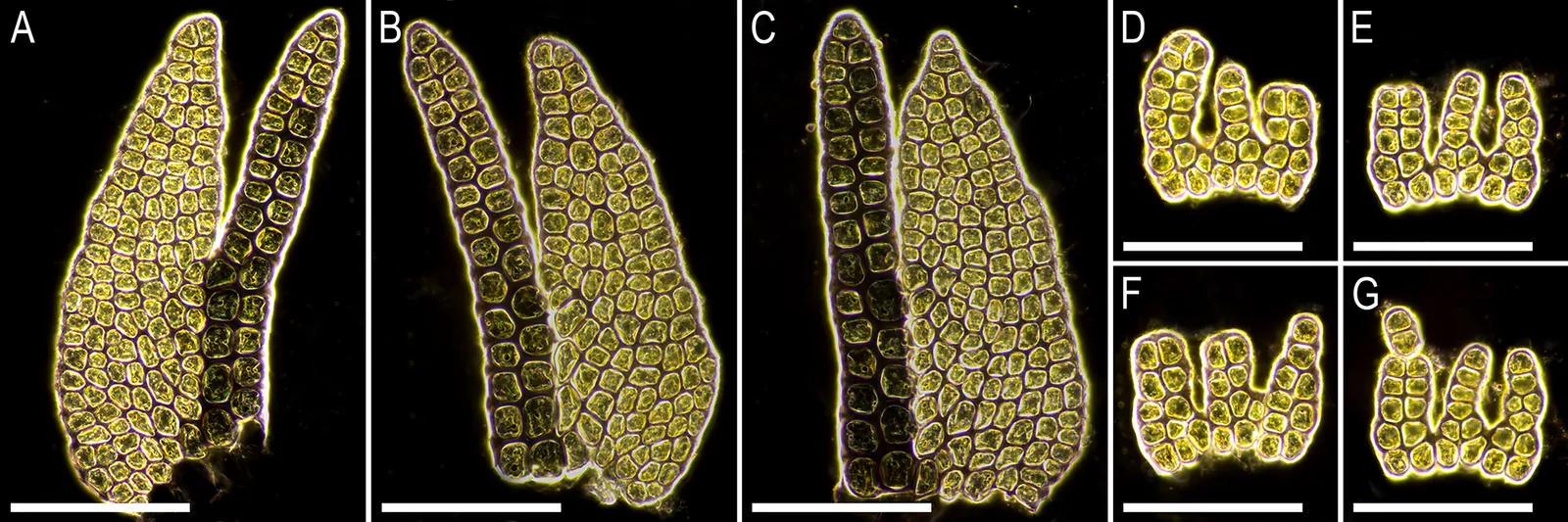
*Acromastigum herzogii* Grolle. (A–C) Leaves. (D–G) Underleaves. All are photographed with dark field option. Scales: 100 µm for all. All from *V-25-5-23* (VBGI, dupl. in HN).

***Acromastigum herzogii*** Grolle, Oesterr. Bot. Z. 111(2–3): 250. 1964 (Figs 7 and 8)

*Description.* – Plants creeping, loosely attached to the substratum, freely branched and forming loose mat over substrate, including mosses and liverworts, pale green to yellowish green when alive and pale greenish to brownish after several years of keeping in the herbarium, well developed shoots 0.5–0.6 mm wide and 5–15 mm long (older portions decaying and decomposing), branching of the main stem of the *Frullania*-type (modified leaf ovate, unlobed, with acute apex), ventral branching resulting in geotropic positive flagella of *Acromastigum*-type, instead of 1–2 of 3 underleaf lobes (therefore 1–2 lobes remained). Stem in dorsal side with two rows of very thick-walled cells, 40–50 × 15–35 µm, with wall thickness 5–7 µm (from lumen to lumen); cross section slightly transversely ellipsoidal, ca. 90 × 100 µm, with 7–8 outer cells, 20–25 µm in diameter, with thickened walls, with external wall noticeable thickened), trigones large, concave to slightly convex, outer surface smooth; inner cells 3 in number, the same size as outer cells or slightly smaller, thin-walled, trigones large, triangular to slightly convex. Rhizoids very sparse, in terminal portion of the ventral flagella produced from apical cells of the scale-like leaves and underleaves (indistinguishable), colorless, flexuous, 150–300 µm long, separated, obliquely spreading. Leaves contiguous to loosely imbricate, obliquely inserted dorsally and subtransversely in the ventral half, ob-canaliculate, not turned to ventral side even in dry condition, obliquely to substransversely oriented, suberect spreading, obliquely ovate, when flattened in the slide 250–270 × 120–130 µm, divided by V- to narrowly U-shaped sinus into two unequal lobes with dorsal lobe wider and triangular and ventral narrowly lingulate with triangular apex; cuticle scarcely and minutely verrucose, with verrucae 1/5–2.0 µm in diameter. Leaf cells strongly differ in size in ventral and dorsal halves; in the middle of the dorsal half subisodiametric, 7–11 µm in diameter to oblong 10–15 × 6–10 µm, with thickened walls, trigones concave, moderate-sized, cuticle minutely verrucose (rarely almost smooth); in the base of the ventral lobe (actually the lobe is 2 cells wide only) 18–22 µm in diameter, strongly thickened, with moderate in size trigones, cuticle in ventral half lesser distinctly verrucose than in dorsal, although not smooth. Underleaves contiguous to distant, obliquely spreading, not overlapping, nearly the same width as the stem (to slightly narrower or wider) when moistened, when flattened in the slide transversely ellipsoidal, 80–90 × 90–100 µm, divided by narrowly U-shaped sinus descending to 2/3 of the underleaf length into 3 lobes those are narrowly lingulate with rounded apices or triangular with acute apices; cuticle as in leaves. Dioicous (or may be remotely autoicous). Androecia on short ventral branches with 2–3 pairs of sterile leaves in the base and then with 3–4 pairs of bracts, spicate, bracts uniandrous, ventricose, for 1/3 of the length narrowly divided by sinus, bilobed. Archegonia on short ventral branches, with 4 pairs of sterile leaves gradually becoming larger to the 1/2 bilobed and somewhat dentate at the margin bracts.

*Specimens examined.* – VIETNAM: Northeast Vietnam, Phú Thọ Province, Tân Sơn District (before July 01, 2025), Xuân Sơn Commune (Xuân Đài Commune from July 01, 2025), Xuân Sơn National Park, 21.11366°N, 104.93419°E, 1263 m a.s.l., shrubby vegetation on the top of the mountain composed mostly by *Rhododendron* and other evergreen tall shrubs developed over limestone conglomerate, partly shaded moist humus in forest floor, 03 Apr 2023, *V.A. Bakalin & H.M. Nguyen V-21-35-23* (VBGI, dupl. in HN); *ibid.* 21.11762°N, 104.93599°E, 1150 m a.s.l., evergreen tropical forest on steep slope with many rocky outcrops (alkaline, probably limestone conglomerate), partly shaded mesic tree trunk, 03 Apr 2023, *V.A. Bakalin & H.M. Nguyen V-25-5-23* (VBGI); *ibid.* partly shaded moist tree trunk, 03 Apr 2023, *V.A. Bakalin & H.M. Nguyen V-25-6-23* (VBGI); North Central Coast, Nghệ An Province, Tương Dương District, northern part of Annamite Range, Pù Mát National Park, N-facing slope of Pù Mát Mt., 19.02312°N, 104.57874°E, 1552 m a.s.l., dense tropical forest, moist tree trunk base in part shade, 25 May 2022, *V.A. Bakalin & H.M. Nguyen V-36-25-22* (VBGI, dupl. in HN); *ibid.* area near the peak of Pù Mát Mt., 19.02464°N, 104.58004°E, 1613 m a.s.l., dense *Rhododendron* forest, moist tree trunk in part shade, 25 May 2022, *V.A. Bakalin & H.M. Nguyen V-37-16-22* (VBGI); Hà Tĩnh Province, Vũ Quang District (before July 01, 2025), Hương Quang Commune (Quang Thọ Commune from July 01, 2025), northern part of Annamite Range, Vũ Quang National Park, 18.25294°N, 105.33318°E, 1322 m a.s.l., montane evergreen forest on a ridge dominated by Fagaceae, partly shaded moist fallen decaying decorticated tree trunk, 20 Apr 2025, *V.A. Bakalin, K.G. Klimova & M.H. Nguyen V-6-4-25* (VBGI duplicate in HN); Huế City, Phú Lộc District (before July 01, 2025), Lộc Trì Commune (Phú Lộc Commune from July 01, 2025), northern part of Annamite Range, Bạch Mã National Park, trail to the “Rhododendron Falls” on very steep S-facing slope to the bottom of the falls, 16.18618°N, 107.84944°E, 1091 m a.s.l., secondary subtropical evergreen monsoon forest with rocky outcrops and rocks, partly shaded moist humus, 12 May 2025, *V.A. Bakalin, K.G. Klimova & M.H. Nguyen V-49-2-25* (VBGI, duplicate in HN); Central Highlands, Quảng Ngãi Province, Đắk Glei District (before July 01, 2025), Đắk Man Commune (Đăk Plô Commune from July 01, 2025), southern part of Annamite Range, Ngọc Linh Nature Reserve, the top of Ngok Pol Pin Pah Mt., 15.20427°N, 107.71365°E, 1982 m a.s.l., high montane broadleaf evergreen forest with high participation of *Rhododendron* on the ridgeline (‘mossy’ forest), standing decaying trunk, moist, in part shade, 15 May 2025, *K.G. Klimova, V.A. Bakalin & M.H. Nguyen Viet-66-23-25* (VBGI); Lâm Đồng Province, Lạc Dương District (before July 01, 2025), Đạ Chais Commune (Lạc Dương Commune from July 01, 2025), southern part of Annamite Range, Bidoup Núi Bà National Park, southern slope of Hòn Giao Mt., Hòn Giao Ranger Station surroundings. 12.19214°N, 108.71144°E, 1887 m a.s.l., montane evergreen broadleaved humid forest with *Rhododendron*, *Magnolia*, *Podocarpus* on a ridge, partly shaded moist tree trunk 22 May 2025, *V.A. Bakalin, K.G. Klimova & M.H. Nguyen V-68-22-25, V-68-44-25* (VBGI duplicate in HN); *ibid.* nameless mountain 500 meters south of Hòn Giao Ranger Station, 12.18257°N, 108.71700°E, 1843 m a.s.l., montane evergreen broadleaved forest on the ridgeline with small admixture of *Fokienia hodginsii*, standing decaying trunk, moist, in part shade, 24 May 2025, *K.G. Klimova, V.A. Bakalin & M.H. Nguyen Viet-86-12-25* (VBGI duplicate in HN); THAILAND: Loey, Phu Luang Mt., 1500 m a.s.l., tree trunk in moist evergreen forest, 05 Dec 1965, *M. Tagawa & N. Kitagawa T1673* (NICH 277677, det. N. Kitagawa: *Acromastigum divaricatum* (Gottsche, Lindenb. & Nees) A. Evans; dupl. in VBGI); Prachinburi, Khao Yai, Khao Khieo, 1100 m a.s.l., tree trunk in moist evergreen forest, 16 Feb 1966, *A. Touw 12173* (NICH 297393, det. N. Kitagawa: *Acromastigum angustilobum* N. Kitag., note: *nomen herbariorum*; dupl. in VBGI); Udawn, Phu Luang Mt., 1350–1400 m a.s.l., on trees in dense low evergreen forest near streamlet, 09 Jan 1966, *A. Touw 10671* (NICH 297486, det. N. Kitagawa: *Acromastigum angustilobum* N. Kitag., note: *nomen herbariorum*; dupl. in VBGI).

*Ecology.* – The species seems to occur in various habitats, including living and fallen mostly moist tree trunks and mesic humus between 1000 and 2000 m a.s.l. in mountain south subtropical forest communities distributed above the tropical forest stand rarely enters to tree *Rhododendron* dominating forests in the ridgelines. The ecological data provided in Pócs & al. [20] and Pócs [21] correspond to those we observed. The ecology briefly described in the specimen citation by Grolle [11] is also similar to our observation, except for the somewhat lower diapason in Borneo, where the species was found at an elevation of ca. 800 m a.s.l.

*Distribution.* – The species was first recorded from Vietnam by Pócs & al. [20] from three locations in Bidoup-Núi Bà National Park (Lâm Đồng Province) in mixed and coniferous forests at altitudes ranging from 1480 to 1700 m a.s.l. (all from decaying logs and bark). Later, Pócs [21] reported the same species from the same province on the basis of the P. Tixier collection (“Benom da Treu, s.n. (PC[PC0764741])”). The general distribution of the species is still poorly known. The species is known from China (Guangxi Province), Malaysia (Sarawak), Thailand, and Vietnam [11,15,20].

In reporting *Acromastigum herzogii* for Vietnam for the first time, Pócs & al. [20] (2019) noted (l.c., p. 401) “A plant published and illustrated by Kitagawa [13, figure 2 in l.c.] from Thailand under the name of *Acromastigum curtilobum* A. Evans may be the same species”. Kitagawa [13] does indeed provide a drawing and a brief note on this species, noting that he collected an atypical form of *A. curtilobum*, distinguished from typical ones by its nonfragile leaves. Notably, the difference between the two species lies not only in the fragility of the leaves but also in the unique leaf attachment in *A. herzogii*, which curves from the dorsal to the ventral side, where in the ventral half, the insertion line becomes transverse. Kitagawa [13] cites three specimens of *A. curtilobum*, two of which (Touw 10671 and Touw 12173) were examined by us at NICH. Both envelopes are labeled “*Acromastigum angustilobum* N. Kitag.”—a name that has never been validly published. This is likely the reason for Kitagawa’s [13: page 50] remark, “However, I am not inclined to describe a new taxon for them”. The plants fully correspond to the morphotypes of *A. herzogii*. Thus, the report of *A. curtilobum* for Thailand should be replaced for *A. herzogii*. Moreover, when working at NICH, we were able to find a specimen labeled *A. divaricatum* (leg. by Tagawa & Kitagawa T1673), which seems to have never been published. The sample’s plants also belongs to the rather typical *A. herzogii*.

*Comment.* – If to formally use the identification key for the genus *Acromastigum* in the revision by Evans [7], it is easy to arrive at *A. divaricatum*. *Acromastigum divaricatum* is somewhat similar to *A. herzogii* in the structure of the stem in cross section; the shape of the leaf; and, in part, the cellular structure of the leaf, with a narrow ventral lobe composed of larger cells. However, the basic difference lies in the position of the leaf insertion line, which undergoes curvature from oblique in the dorsal part to almost transverse in the ventral part. This curvature of the insertion line in other species of *Acromastigum* provided the basis for Evans [7] to describe a special section within the genus *Acromastigum* (sect. *Subcomplicata* Evans). However, recent data [1] do not support the taxonomic significance of this character, and of the two species assigned to it by Grolle [11], one (*Acromastigum filum* (Steph.) A.Evans) was referred to subg. *Inaequilatera*, and the second (*A. herzogii*) to subg. *Acromastigum*. Interestingly, our data on a limited material (only *trn*G) show that *A. herzogii* belongs to subg. *Inaequilatera*, where the voucher specimens are situated in a sister position to the clade shared by *Acromastigum divaricatum* and *A. cunninghamii* (Steph.) A. Evans. However, the scarce data do not allow us to judge phylogenetic relationships with sufficient certainty.

Grolle [11] treats *Acromastigum herzogii* as the most similar to *A. filum* and *A. capillare*. *Acromastigum herzogii* shares primarily its transverse leaf insertion and grooved leaves with *A. filum*, whereas *A. capillare* is similar in leaf cell network features and unequal leaf lobes. Grolle [11] describes the differences between *A. herzogii* and *A. capillare* in 1) underleaves as long as wide to longer than wide (however, in our case, underleaves are slightly wider than long), 2) longer underleaf lobes, with the middle lobe usually 4 cells long, 3) wider dorsal lobe, 4) ventral lobe longer than the dorsal lobe (versus subequal in length), 5) distinctly curved dorsal margin at the base and 6) nearly transverse leaf insertion (actually, the insertion line experiences the curve from oblique to transverse in the ventral half). *Acromastigum herzogii* differs from *A. filum* by essentially the same features, except for leaf insertion (which is similar in both taxa) and the less deeply divided leaves.

***Acromastigum inaequilaterum*** (Lehm. et Lindenb.) A.Evans, Ann. Bryol., Suppl. 3: 129. 1934

**Fig 9:**
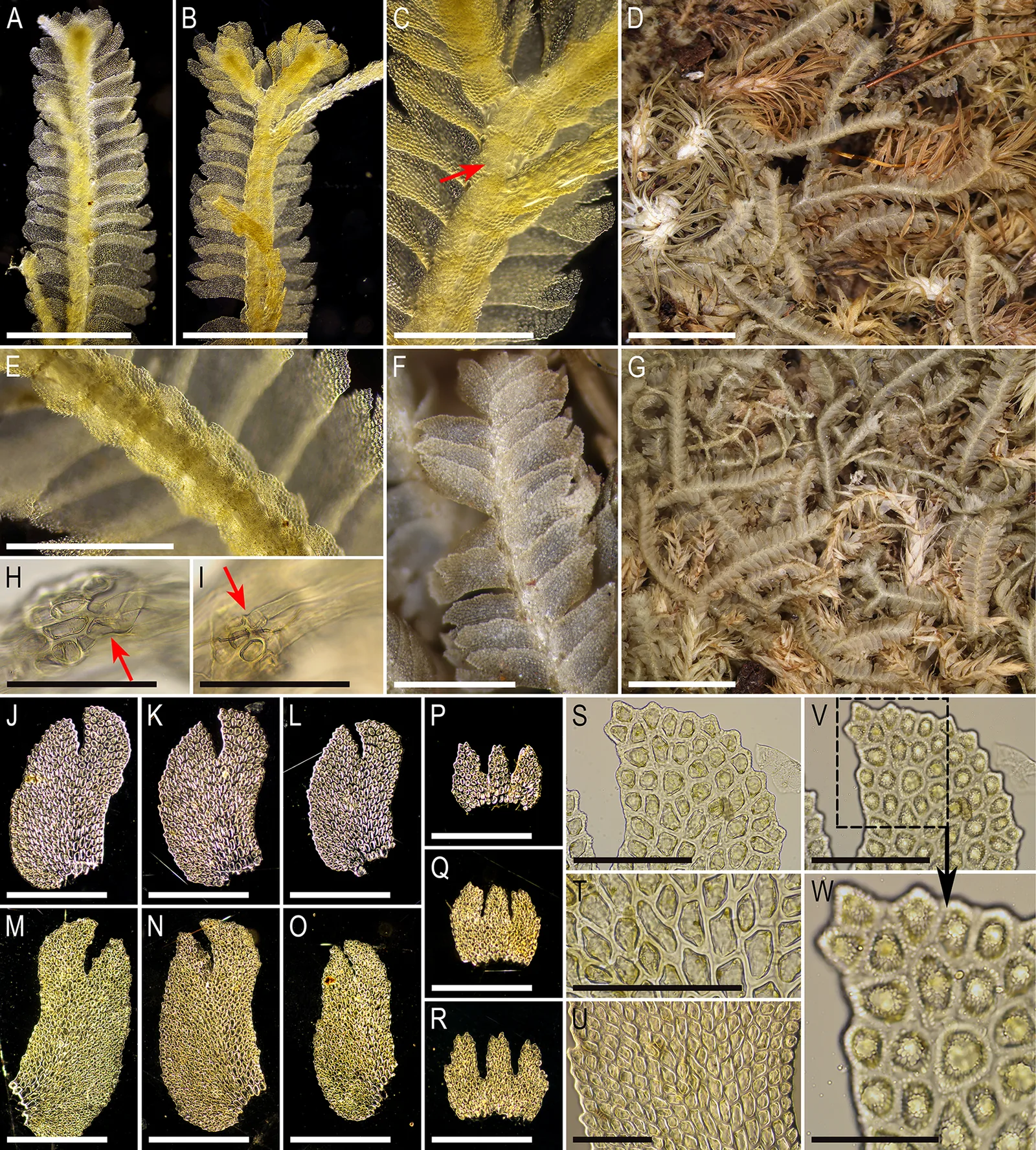
*Acromastigum inaequilaterum* (Lehm. et Lindenb.) A. Evans. (A) Shoot with ventral flagella, dorsal view. (B) Shoot with ventral flagella, ventral view. (C) Fragment of shoot with 1-lobed underleaf at the flagella base indicated by red arrow, ventral view. (D, G) Parts of mats in dry condition. (E) Underleaves on the stem, fragment, ventral view. (F) Upper part of the shoot in dry condition, dorsal view. (H, I) Rhizoids produced from apical cells of the scale-like leaves and underleaves on ventral flagella. (J–O) Leaves. (P–R) Underleaves. (S) Cells of leaf lobe. (T) Midleaf cells. (U) Cells of the leaf middle part. (V) Cells of leaf lobe with verrucae distributed above the cell lumen, covered with papillae. (W) Enlarged portion of V. A–C, E, J–R, U are photographed with dark field option. Scales: 2 mm for D, G; 1 mm for A, B; 500 µm for C, E, F; 300 µm for J–R; 100 µm for H, I, S–V; 50 µm for W. A–D, J–L, P, I, S–W from *Cam-84-16-11* (VBGI); E from *Cam-84-15-11* (VBGI); H, M–O, Q, R from *Cam-84-17-11* (VBGI); F, G from *Cam-85-24-11* (VBGI).

**Fig 10:**
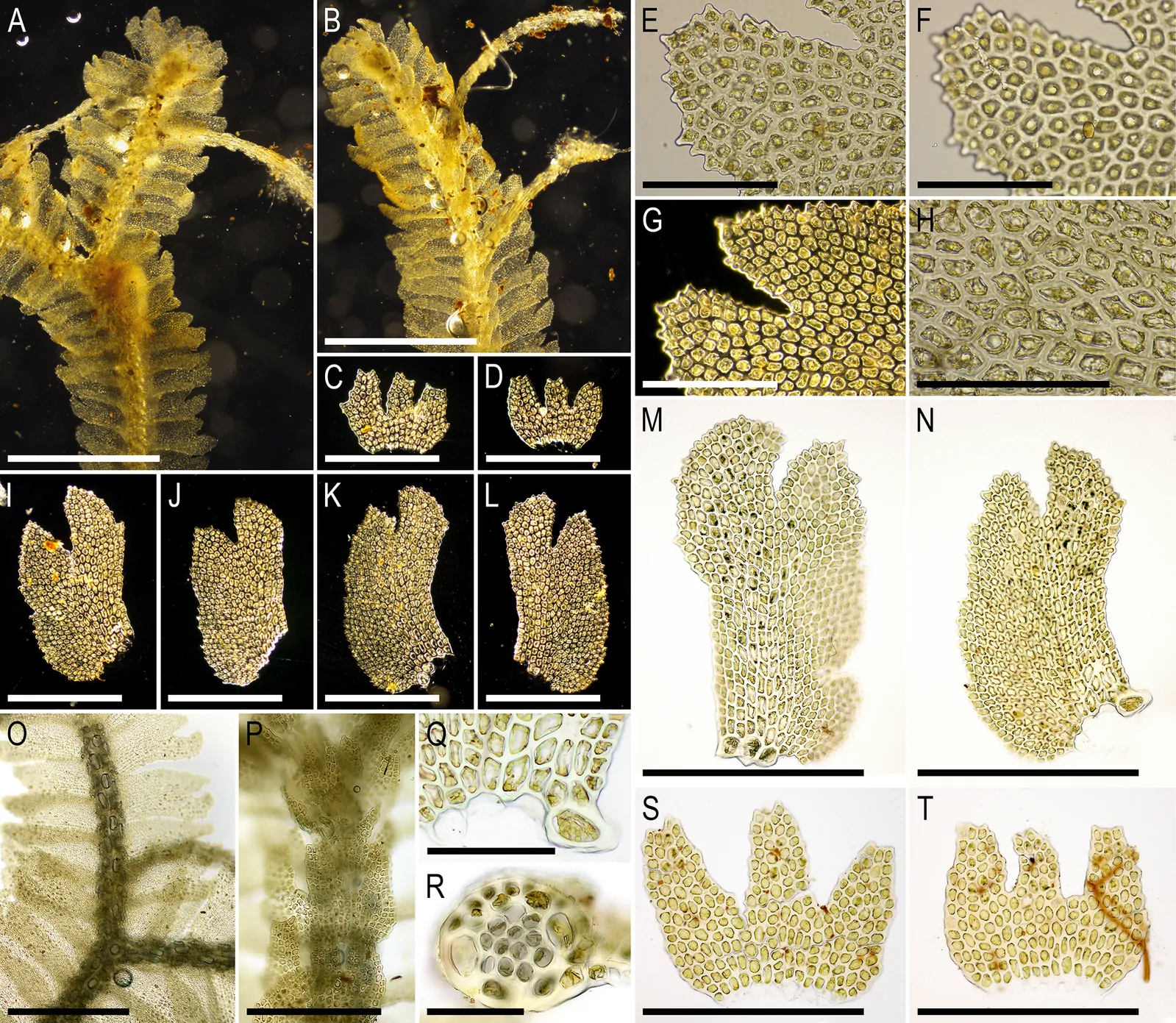
*Acromastigum inaequilaterum* (Lehm. et Lindenb.) A. Evans. (A) Shoot with ventral flagella, dorsal view. (B) Shoot with ventral flagella, ventral view. (C, D, S, T) Underleaves. (E, G) Cells of leaf lobes. (F) Cells of leaf lobes with verrucae distributed above the cell lumen. (H) Midleaf cells. (I–N) Leaves. (O) Fragment of the shoot with large epidermal cells of the stem, dorsal view. (P) Underleaves on the stem, fragment, ventral view. (Q) Cells of leaf base. (R) Stem cross-section. A–D, G, I–L are photographed with dark field option. Scales: 1 mm for A, B; 500 µm for O; 300 µm for C, D, I–N, S, T; 100 µm for E–H, R; 50 µm for Q. A–L from *W. Meijer 4975* (NICH 216292Ok!); M–T from *S.S. Choi C9299* (JNU).

≡*Jungermannia inaequilatera* Lehm. & Lindenb., Nov. Stirp. Pug. 6: 56. 1834 (Figs 9 and 10)

*Description.* – Plants prostrate, loosely attached to the substratum, freely branched and forming loose mats, pale green when fresh and yellowish brownish when dry in herbarium after 10 years of keeping, fragile when dry (commonly loose the ventral flagella), 0.7–1.0 mm wide and 5–15 mm long (older parts decaying and decomposing), branching of the main stem of *Frullania*-type (modified leaf ovate in general outline, unlobed, acute at apex), ventral branching resulting in geotropic positive flagella of *Acromastigum*-type, instead of 1–2 of 3 underleaf lobes (therefore 1–2 lobes remained). Stem cross section slightly transversely ellipsoidal, ca. 125 × 145 µm, with 7–8 outer cells 25–38 × 30–58 µm, with thickened walls, outer wall noticeable thick, trigones large, concave, outer cuticle smooth; inner cells 12–18 × 18–25 µm, with slightly thickened walls and large, triangle to slightly convex trigones. Rhizoids scattered in the terminal portions of flagella, colorless, flexuous, 150–300 µm long, originating from the terminal cells of scale-like leaves or underleaves (indistinguishable), separated, obliquely spreading. Leaves imbricate, covering ca. 1/3–1/2 of the above situated leaf in its basal part, obliquely inserted and oriented, suberect spreading, distinctly turned to the ventral side, convex, when flattened obliquely ovate, unequally bilobed, 500–550 × 275–300 µm in well-developed shoots, divided by V- to U-shaped sinus descending to 1/5–1/4 of the leaf length (actually difficult to measure, because lobes are unequal in the length) into two strongly unequal lobes with rounded apices, dorsal lobe distinctly smaller, leaf margin distinctly dentate at least in the upper halves of the leaves. Midleaf cells 12–25 × 12–18 µm, thick-walled, distinctly papillose (longer cells) to verrucose (subisodiametric cells), verrucae distribute above the cell lumen, while the papillae may stretch across the cell walls; becoming distinctly smaller to the margins, near dorsal margin subisodiametric, 10–15 × 12–18 µm, with thick walls, strongly verrucose with verrucae restricted to the central part of the cell lumen, additionally the cells are mamillosely projecting above the surface. Underleaves transversely ellipsoidal loosely appressed to the stem commonly covering the lower part of the above situated underleaf, divided into 3 subequal lobes by the V- to U-shaped sinus descending to 1/2 of the underleaf length, lobe apices rather obtuse to slightly emarginate, when flattened in the slide 150–175 × 200–225 µm, surface prominently papillose above the cell lumen, but not above the cell walls, lobe margins commonly distinctly dentate.

*Specimens examined.* – CAMBODIA: Koh Kong Province, Thma Bang Village area, 11.68750°N, 103.42722°E, 400 m a.s.l., broadleaved evergreen forest on slope to waterfall, decaying wood, 23 Dec 2011, *V.A. Bakalin Cam-84-15-11, Cam-84-16-11, Cam-84-17-11* (VBGI); the national Mega Forest Area, 11.62583°N, 103.26861°E, 200 m a.s.l., broadleaved evergreen forest on slope, decaying wood, 24 Dec 2011, *V.A. Bakalin Cam-85-20-11, Cam-85-21-11, Cam-85-22-11, Cam-85-24-11* (VBGI); *ibid.* 11.58750°N, 103.19139°E, 200 m a.s.l., broadleaved evergreen forest on slope, stone side, 24 Dec 2011, *V.A. Bakalin Cam-86-3-11* (VBGI); Thma Bang District, near Stoeng Kaoh Pao, 11.696444°N, 103.116028°E, 289 m a.s.l., stream side in the forest, wet, 9 May 2020, *S.S. Choi C9299* (JNU); INDONESIA: Borneo, East Kalimantan, Kutai Kartanegara Regency, Samboja Karya, Merdeka, Bukit Bangkirai, 1.02524°S 116.86486°E, 100 m a.s.l., on rotten log near the stream, 19 Jul 2002, *T. Yamaguchi s.n.* (VBGI); *ibid. T. Yamaguchi s.n.* (NICH 239584!; dupl. in VBGI); East Borneo, Nov 1953, *W. Meijer 4975* (NICH 216292!).

*Ecology.* – Similarly to *Acromastigum echinatum* in East Indochina, *A. inaequilaterum* is the species of lower elevations, not ascending above 400 m a.s.l. The species prefers mesic habitats including fallen decaying decorticated tree trunks and the sides of stones and was once found on wet humus on a stream bank; all were in semi-evergreen (some trees with deciduous leaves in the dry season) forests. The ecology provided for New Guinea by Piippo [10] is similar in elevation measurements but (where known) is restricted to tree trunks [of living trees(?)—not indicated in the original source]. The specimen from Indonesia cited in “Specimens examined” came from a rotten log near the stream, as indicated on the label.

*Distribution.* – This species was reported in Cambodia from Kampot and Koh Kong Provinces [25,26]. Its general distribution extends from Nepal through Indochina (Cambodia) and Malesia to Melanesia [9,48]. The spatial relationships of the closely related *A. inaequilaterum* and *A. echinatum* are unclear; it is possible that both taxa are distributed sympatrically in Koh Kong Province, Cambodia. One of the specimens listed here (*C9299*) was reported by Bakalin et al. [27] as *A. divaricatum*.

*Comment.* – A discussion on differentiation from morphologically similar taxa is provided under *A. echinatum*. The additional illustrations and descriptions for this species are provided by Evans [7] and Grolle [48].

**Fig 11:**
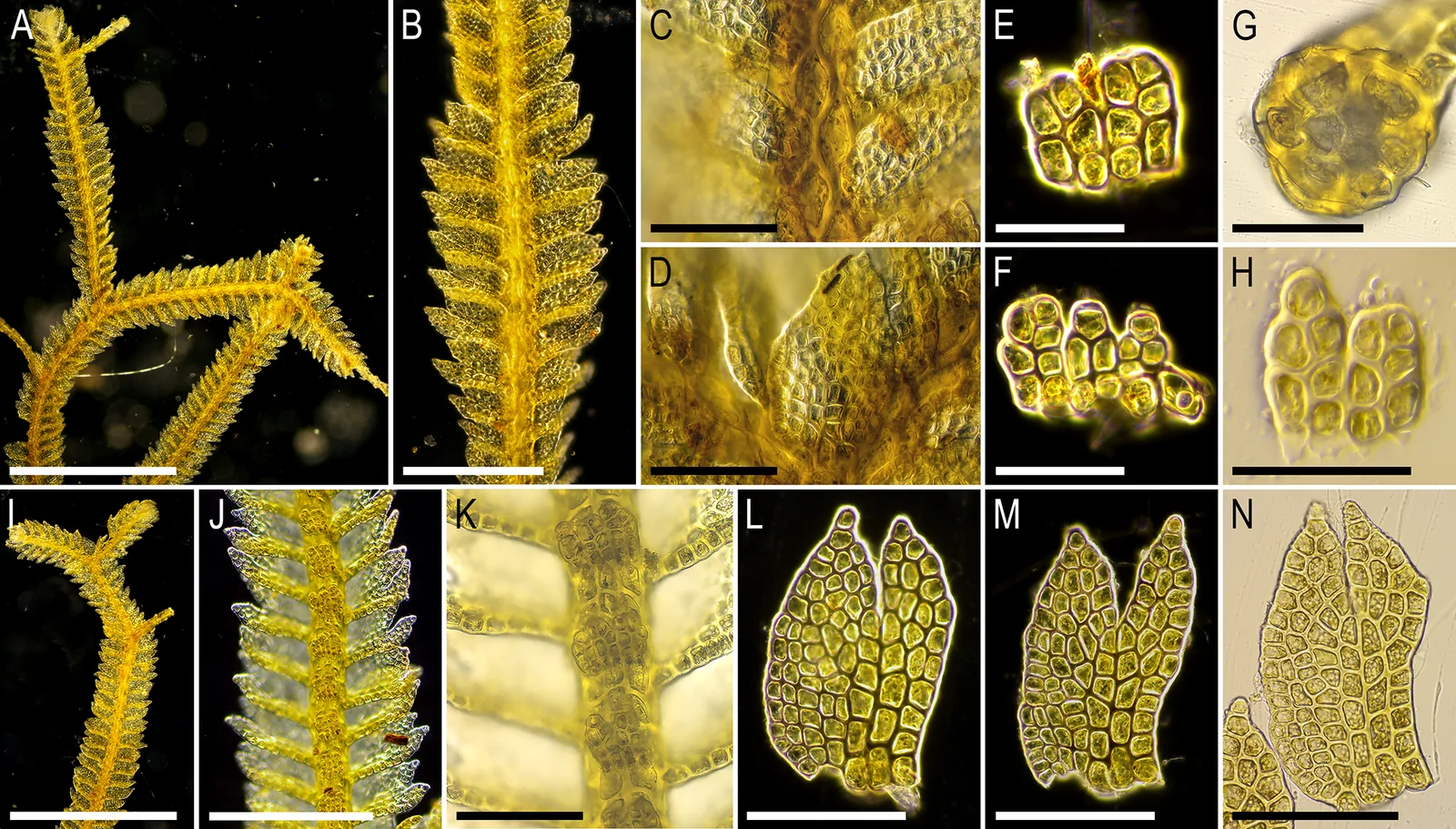
*Acromastigum laevigatum* A. Evans. (A) Shoot with ventral flagella, dorsal view. (B) Fragment of the shoot middle part, dorsal view. (C) Oblong cells of dorsal side of the stem. (D) Terminal branching of the main shoot of the *Frullania*-type with covering ovate-lanceolate leaf with acute apex. (E, F, H) Underleaves. (G) Stem cross-section. (I) Upper part of the shoot with ventral flagella, ventral view. (J) Fragment of the shoot middle part, ventral view. (K) Underleaves on the stem, fragment, ventral view. (L–N) Leaves. A–F, I, J, L, M are photographed with dark field option. Scales: 1 mm for A, I; 300 µm for B, J; 100 µm for C, D, K–N; 50 µm for G, H; 30 µm for E, F. All from *Choi C9013* (JNU, dupl. in VBGI).

***Acromastigum laevigatum*** A.Evans, Ann. Bryol., Suppl. 3: 101. 1934 (Fig 11)

*Description.* – Plants prostrate, loosely attached to the substrate, yellowish brownish to greenish brownish, forming small pure patches, 0.35–0.4 mm wide and 3–6 mm long (older parts decomposing), terminal branching of the main shoot of the *Frullania*-type with covering leaf ovate-lanceolate with acute apex, ventral branching leading to ventral geotropic flagella of *Acromastigum*-type accompanying by unilobed underleaf. Stem in dorsal side with oblong unclearly ellipsoidal cells 45–55 × 25–30 µm, thick-walled, with walls (from lumen to lumen) ca. 5 µm thick; stem cross section nearly rounded, 60–70 µm in diameter, with 6 outer cell rows, outer cells 18–20 µm in diameter, thick-walled except of the innermost wall, outer stem wall with smooth surface; inner cells in three rows, 12–15 µm in diameter, nearly thin-walled, with moderate in size to small trigones. Rhizoids rare, at the terminal portions of the ventral flagella, colorless, flexuous, obliquely spreading, separated or in unclear fascicles, 150–200 µm long. Leaves obliquely inserted with insertion line shortly but distinctly curved to subtransverse in its ventral end, obliquely oriented, obcanaliculate, obliquely spreading, not turned to the ventral side, somewhat imbricate and covering ca. 1/4 of the above situated leaf; when flattened in the slide the well-developed leaf 160–200 × 100–120 µm, divided by V- to ɣ-shaped sinus descending 1/3–2/5 of the leaf length into two subequal (ventral looks larger in the wetted plants) acute triangular lobes. Leaf cells nearly the same size in dorsal and ventral halves, in the middle part rectangular to irregularly oblong, 15–20 × 12–15 µm, thick-walled, trigones small, concave, to the dorsal margin smaller, 7–13 × 7–10 µm, thick-walled, external wall only slightly thickened, cuticle smooth to scarcely verrucose. Underleaves narrowly obliquely spreading, distinctly narrower than the stem, nearly transversely ellipsoidal, 40–50 × 50–65 µm, shortly trilobed by V-shaped sinus descending to 1/4–1/3 of the underleaf length, with lobed triangular to lingulate, 1–2 cells long and 1–2 cells wide; commonly becoming bilobed in depauperate shoots.

*Specimens examined.* – CAMBODIA: Kampot Province, Elephant Mountains, Dreah Monivong Bokor National Park, 10.62333°N, 104.07278°E, 992 m a.s.l., forest roadside, 4 May 2010, *S.S. Choi C9013* (JNU, dupl. in VBGI).

*Ecology.* – Our collection cited in the specimens examined comes from the manmade habitat (roadside) and represents depauperate plants (may be to the latter reason). It was collected near 1000 m a.s.l. in an evergreen tropical mountain forest. The literature report for Vietnam by Jovet-Ast & Tixier [24] comes from a thick trunk base in dense forest at an altitude of approximately 1500 m a.s.l. In Malaysia [45: page 22], it is cited as “corticolous in forest, 900 m”.

*Distribution.* – The species was recorded in Vietnam from Măng Lin [‘Manline’ in l.c.] in the vicinity of Đà Lạt City. In addition to the abovementioned occurrence in Vietnam [24], the species is found in Cambodia (the present paper) and in Malaysia (Kedah, Sarawak) [7,9,24,50,51].

*Comment.* The description and illustrations of the species are provided by Evans [7]. The species is characterized by obliquely inserted, slightly unequally bilobed leaves and is most similar to *A. divaricatum*. *Acromastigum laevigatum* differs from *A. divaricatum* at first glance in that the ventral lobe is shorter than the dorsal lobe (the opposite is true in *A. divaricatum*). Evans [7] described the differences between the two species in detail. In addition to the abovementioned difference in lobe length, the following is noticeable: 1) (l.c., 106) “the texture of *A. laevigatum*, however, is firmer than that of *A. divaricatum*, the tissues are less transparent, and the leaves are relatively broader, so that their shape is ovate rather than ovate-rectangular”; 2) “in *A. divaricatum* the ventral margin appears in surface view when a branch is examined from below” versus “the ventral margin throughout more or less of its extent appears in profile view” in *A. laevigatum*; 3) “the leaf-cells in most cases show distinct trigones separated by thin areas” versus “the leaf-cells rarely if ever show distinct trigones” in *A. laevigatum*. In connection with the above (the sharp bend of the insertion line at its ventral end), it may be difficult to distinguish *A. laevigatum* from *A. herzogii*. However, differentiation of both is easily possible during leaf preparation. In *A. herzogii*, the cells in the ventral lobe are clearly larger than those in the dorsal lobe, and the width of the lobe is made up of fewer cells (usually 2 cells only), whereas in *A. laevigatum*, the cells are similar in size in both lobes, and their number across the width in the ventral lobe is greater than that in *A. herzogii*. Even in our likely depauperate plants, it is never less than three (Evans, [7], indicates 4–5). Notably, Evans [7] reported 7 rows of cells in the inner layer of the stem in cross section, but we observed only 3 rows of cells. This is probably because our plants belong to a small suppressed form of the species, which is also suggested by the predominance of bilobed underleaves in the vast majority of studied plants. Additionally, it is worth noting the striking difference in the appearance of the plants in the dark-field photographs. In *A. herzogii*, the ventral lobe is dark and barely glistening, whereas in *A. laevigatum*, it does not differ in reflected light intensity from the dorsal lobe (as in other studied species).

**Fig 12:**
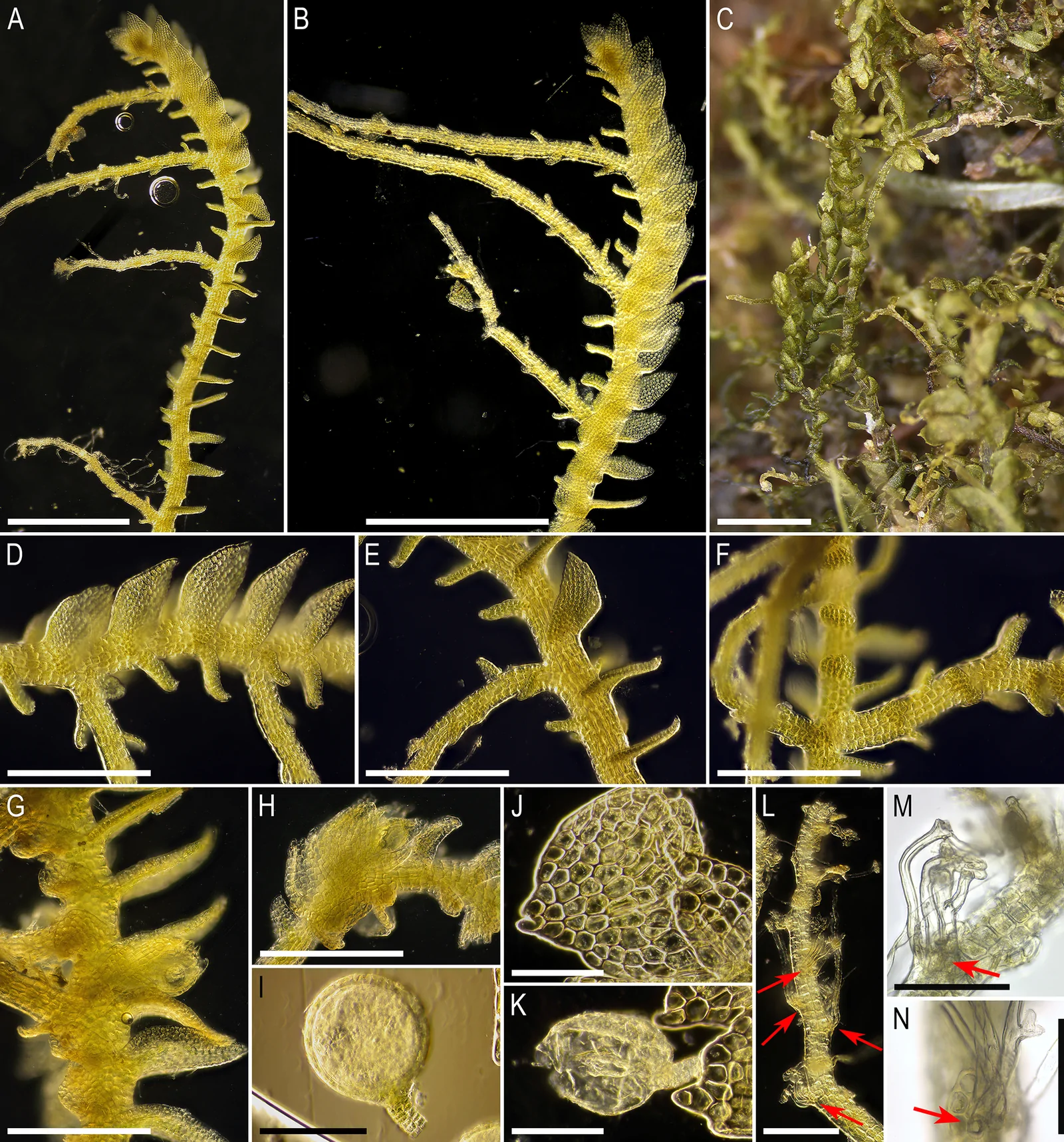
*Acromastigum vietnamicum* Bakalin, Klimova, Maltseva sp. nov. (A, B) Shoots with ventral flagella, lateral view. (C) Plants in a mat in dry condition. (D, E) Fragment of the shoot middle part, a row of leaves, a row of underleaves, and ventral flagella are visible, lateral view. (F) Fragment of the shoot middle part, a row of underleaves and ventral flagella are visible, ventral view. (G) Fragment of the shoot middle part with androecia on short ventral branch, ventral view. (H) Fragment of the shoot middle part with androecia on short ventral branch, lateral view. (I) Antheridium. (J) Antheridial bract. (K) Opened antheridium. (L) Ventral flagella with rhizoid fascicles from the basal cells of the scale-like leaves and underleaves, indicated by red arrows, ventral view. (M, N) Rhizoids originated from the basal cells of the scale-like underleaves. A, B, D–L are photographed with dark field option. Scales: 1 mm for A–C; 500 µm for D–H; 200 µm for L; 100 µm for I–K, M, N. A–F, L–N, from *Viet-80-11-25* (VBGI); G, H from *V-69-5-25* (VBGI); I–K from *V-69-4-25* (VBGI).

**Fig 13:**
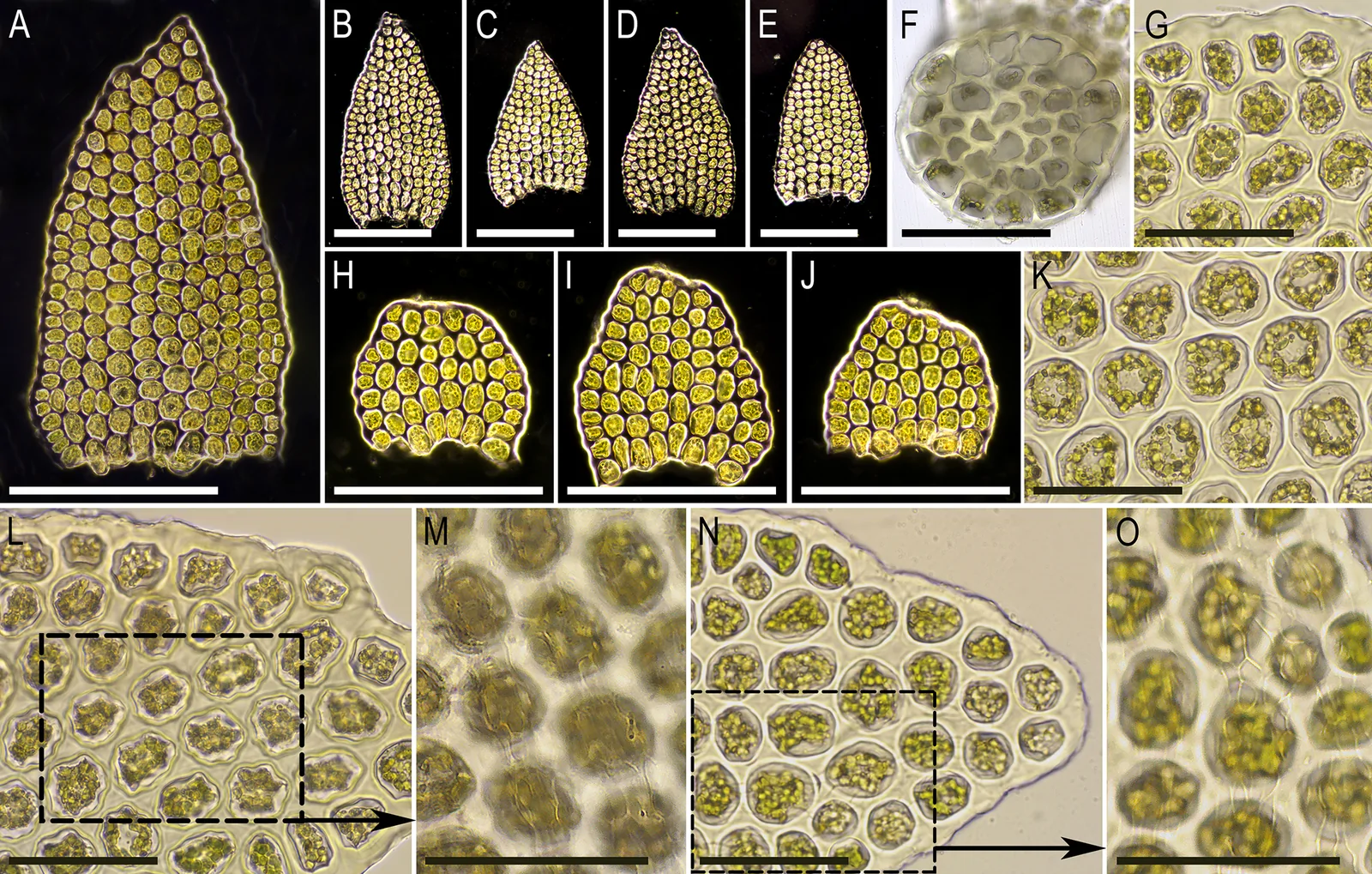
*Acromastigum vietnamicum* Bakalin, Klimova, Maltseva sp. nov. (A–E) Leaves. (F) Stem cross-section. (G) Cells of leaf margin. (H–I) Underleaves. (K) Midleaf cells. (L, N) Cells of leaf apex. (M) Enlarged and rotated 90 degrees counterclockwise portion of L. (O) Enlarged and rotated 90 degrees counterclockwise portion of N. A–E, H–J are photographed with dark field option. Scales: 200 µm for A–E, H–J; 100 µm for F; 50 µm for G, K–O. All from *Viet-80-11-25* (VBGI).

***Acromastigum vietnamicum*** Bakalin, Klimova, Maltseva **sp. nov.** – Holotype: Vietnam, Central Highlands, Lâm Đồng Province, Lạc Dương District (before July 01, 2025), Đạ Chais Commune (Lạc Dương Commune from July 01, 2025), southern part of Annamite Range, Bidoup Núi Bà National Park, the top of Hòn Giao Mt., 12.21140°N, 108.71872°E, 2003 m a.s.l., dense mountain evergreen crooked humid ‘mossy’ forest with young *Rhododendron* trees (about 3 meters high), Fagaceae trees and bamboo, partly shaded moist fallen decaying decorticated tree trunk, 23 May 2025, *V.A. Bakalin, K.G. Klimova & H.M. Nguyen V-69–5–25* (VBGI, dupl. in NH). (Figs 12 and 13)

*Description. –* Plants prostrate to ascending, forming pure mats or intermixed with other liverworts and/or mosses, loosely attached to the substrate, yellowish greenish when fresh and greenish yellowish in the herbarium after one year of keeping plants subisophyllous, 0.4–0.6 mm wide and 4–15 mm long (may be longer, but fragile when taking off the substrate). Terminal branching of *Frullania*-type not seen, while the both ventral geotropic positive flagella and normal branches produced from the ventral branches of *Acromastigum*-type, adjacent underleaf becoming narrower (the branch is instead of the ‘half’ of the underleaf), but at least in the several cases the corresponding underleaf is completely absent; branches sometimes paired. Stem cross section orbicular or nearly so, ca. 150 µm in diameter, outer cells in 13–14 rows, 25–35 µm in diameter, thin-walled, but external wall strongly thickened, trigones large, convex, outer cuticle smooth; inner in 16–20 rows, somewhat smaller than outer, 15–20(–25) µm in diameter, thin-walled, trigones large, triangular to concave or slightly convex. Rhizoids from the basal cells of the scale-like leaves and underleaves (indistinguishable) in the terminal part of the flagella, separated or in loose fascicles, obliquely spreading, flexuous, 200–400 µm long, mostly densely divaricately branched at the terminal areas. Leaves distant, rarely contiguous, transversely inserted, transversely or nearly so spreading and oriented that incubous nature is commonly not evident, nearly plane to slightly convex, with apices unclearly turned to the stem apex; when flattened in the slide ovate to ovate-triangular, obtuse to subacute, or sometimes with unclearly and obliquely emarginate apex, 320–450 × 180–240 µm, somewhat crenulate along margin (*Allorgella*-type denticulations). Midleaf cells subisodiametric, 20–30 µm in diameter, thin-walled, trigones large, sometimes loosely confluent, cuticle smooth to very unclearly papillose; to the margin becoming smaller, subquadrate, 12–18(−25) µm in diameter, thick-walled, trigones large, mostly concave, external wall strongly thickened and trigones adjacent to it convex, radial cell walls protrudent over the margin line and forming *Allorgella*-type denticulations, cuticle smooth or the nearest to the margin areas and unclearly verrucose. Underleaves erect to obliquely spreading, distant, sometimes slightly convex (when looking from the ventral side), reniform to spoon-shaped, 140–220 × 150–200 µm, with apex unclearly emarginate. Seems dioicous (gynoecia not seen). Androecia on short ventral branches, with 1–2 pairs of sterile leaves in the base and then with 2–3 pairs of bracts (actually may be longer, but not yet observed), uniandrous, body ca. 140 µm in diameter, stalk biseriate, 20–30 µm long; bracts widely ovate, at apex acute to shortly bilobed.

*Other specimens examined (paratypes).* – VIETNAM: Central Highlands, Lâm Đồng Province, Lạc Dương District (before July 01, 2025), Đạ Chais Commune (Lạc Dương Commune from July 01, 2025), southern part of Annamite Range, Bidoup Núi Bà National Park, southern slope of Hòn Giao Mt., Hòn Giao Ranger Station surroundings, 12.19214°N, 108.71144°E, 1887 m a.s.l., montane evergreen broadleaved humid forest on a ridge, decaying log leaning over the ground, moist, in part shade, 22 May 2025, *K.G. Klimova, V.A. Bakalin & H.M. Nguyen Viet-80–10–25, Viet-80–11–25, Viet-80–12–25* (VBGI, dupl. in HN); *ibid.* the top of Hòn Giao Mt., 12.21140°N, 108.71872°E, 2003 m a.s.l., dense montain evergreen crooked humid ‘mossy’ forest with young *Rhododendron* trees (about 3 meters high), Fagaceae trees and bamboo, partly shaded moist fallen decaying decorticated tree trunk, 23 May 2025, *V.A. Bakalin, K.G. Klimova & H.M. Nguyen V-69–4–25* (VBGI, dupl. in NH); *ibid.* ridgeline of southern slope of Hòn Giao Mt., 12.20729 °N, 108.71734 °E, 1983 m a.s.l., montane evergreen broadleaved humid scattered forest on the ridgeline with dense bamboo understory, partly shaded moist fallen decaying decorticated tree trunk, 23 May 2025, *V.A. Bakalin, K.G. Klimova & H.M. Nguyen V-71-3-25* (VBGI, dupl. in HN).

*Ecology.* – *Acromastigum vietnamicum* was found in montane evergreen broadleaved humid forests on fallen decaying tree trunks, decorticated or not, and grew in mixed mats accompanied by species of *Bazzania, Cheilolejeunea, Herbertus, Lepidozia, Plagiochila* and *Riccardia.*

*Distribution.* – Hitherto known from authentic material only.

*Comment.* – The group of subisophyllous *Acromastigum* species with transversely inserted and entire or very short-lobed leaves includes *A. caledonicum* (Steph.) Grolle, *A. homodictyon* (Herzog) Grolle, *A. verticale* (Steph.) E.A. Hodgs., *A. cavifolium* R.M. Schust. and *A. integrifolium* (Austin) A.Evans. Of these, *A. caledonicum* differs from *A. vietnamicum* in its uniformly thickened leaf and underleaf cell walls, the presence of a vitta-like structure along the midline of the leaf and a stem cross section with fewer rows of cells in both the outer and inner layers. *Acromastigum integrifolium* resembles *A. vietnamicum* in stem cross-section but is characterized by ovate, non-pointed leaves that are indistinctly emarginate at the tips. Furthermore, the cuticle of this species is coarsely papillose, and the leaves are convex rather than concave [48]. *Acromastigum homodictyon* resembles *A. vietnamicum* in the shape of the underleaves (including their width not exceeding that of the stem, i.e., slightly narrower than that of *A. vietnamicum*), but differs in its ovate leaves with a blunt, shortly divided apex and a unique stem cross-section, where the boundary between the outer and inner layers is not clearly defined.

Among the abovementioned species, *Acromastigum cavifolium* (differentiation from which has been assessed genetically) and *A. verticale* are apparently the most morphologically similar to *A. vietnamicum*. Both of these species are characterized, in addition to subisophyllous shoots, by entire, bluntly pointed to vaguely (very shortly) bilobed leaves. *Acromastigum verticale* has somewhat falcate leaves, clearly distinguishing it from *A. vietnamicum*. The morphological similarity to *A. cavifolium* is much greater, and quantitative rather than qualitative traits should be considered. *Acromastigum vietnamicum* is characterized by a much smaller plant size, reaching only 0.6 mm in width (versus 1.0–1.5 in *A. cavifolium*), stem cells in cross-section that are only slightly different from the outer ones (versus sharply different in size), smaller leaf cells, subisodiametric and varying within the range of 20–30 μm in diameter (versus 40–75 × 25–40 μm, cf. [4]), and underleaves that are approximately equal in width to the stem size (versus 1.2–1.7 as wide as the stem).

Key to *Acromastigum* species known in East Indochina:

1. Plants subisophyllous, leaves nearly transversely inserted, ovate with acute apex, unlobed to shortly and obliquely bidentate at the apex, rhizoids from the basal cells of scale-like leaves and underleaves of ventral flagella (subg. *Acromastigum*) … *Acromastigum vietnamicum*
2. Plants anisophyllous, leaves obliquely (at least in dorsal half) incubously inserted, distinctly bilobed, rhizoids from apical cells of the scale-like leaves and underleaves of ventral flagella (subg. *Inaequilatera*) … 2
3. Leaf lobe apices rounded (although may be dentate), lobes somewhat falcate with lobe midline curved toward shoot apex, dense clusters of verrucae are commonly present above the leaf cell lumen (but not above the cell walls) … 3
4. Leaf lobe apices acute, nearly entire to loosely crenulate, lobes not falcate, lobe midline straight, cuticle smooth to papillose, if papillose then papillae distributed across leaf surface including cell lumens and cell walls … 4
5. Leaf margin dentate (rarely entire), midleaf cells with thick walls and owing to the latter unclear and concave trigones … *Acromastigum inaequilaterum*
6. Leaf margin entire to crenulate due to mammillose protrusions of marginal cell walls and clusters of papillae above the cell lumen in marginal cells … *Acromastigum echinatum*
7. The leaf insertion line is curved from oblique in its dorsal half to subtransverse and transverse in ventral half; leaf cells distinctly differ in size between the ventral and dorsal lobes; the ventral lobe is dark and barely ‘glistening’ in dark field photographs … *Acromastigum herzogii*
8. The leaf insertion line does not undergo sharp curvature (although it is slightly curved at the end in *A. laevigatum*), and the leaves are obliquely inserted; leaf cell size in ventral and dorsal lobes subequal; the ventral and dorsal lobes do not differ in reflected light intensity in dark field photographs … 5
9. Cell walls have secondary pigmentation (yellowish to brownish) that is especially obvious in dorsal cortical cells; cells in stem cross section are so thick that the lumen size is the same in inner and outer cells (despite the strong difference in the general diameter measured from mid-lamina to mid-lamina); underleaves wider than the stem and somewhat overlapping; underleaf lobes commonly narrowly lingulate … *Acromastigum echinatiforme*
10. Cell walls lack secondary pigmentation; stem cross section shows distinct cell lumen sizes in outer (larger) and inner (smaller) cells; underleaves distinctly narrower than the stem and distanced (never overlapping); underleaf lobes commonly triangular … *Acromastigum laevigatum*

## Discussion

Six species are known in East Indochina, one of which is described here. We identified five species, and one is cited only on the basis of literature data. Data from Thailand (on the distribution of *A. curtilobum*), discussed under *A. herzogii*, indirectly confirm that some of the literature reports may be referable to other species. However, since we did not study specimens of *A. echinatiforme*, there is currently no basis for excluding it from the flora of East Indochina. Notably, having failed to find firsthand reports for *A. echinatum*, we suspected that its reference to Indochina was erroneous, but it was later discovered in our own collections. This confirms this species for Indochina for the first time on the basis of the cited series of specimens.

One species is described as new to science (*Acromastigum vietnamicum*). This species is also notable because it appears to be the northernmost representative of subg. *Acromastigum*, which is clearly distinct from subg. *Inaequilatera* (if the subgeneric position of *A. herzogii* is doubted). This discovery supports the expectations that other representatives of the genus may be found in Indochina (both new localities of known species and new-for-science taxa). As Schofield [52: page 331] noted, “the study of bryophyte distributions has been somewhat hampered by inadequate documentation. This reflects the limited number of experienced collectors and researchers.” Although this was written more than 40 years ago, the situation has not changed fundamentally and still applies fully to *Acromastigum* in East Indochina; therefore, new discoveries in this genus are looking inevitable.

**Fig 14:**
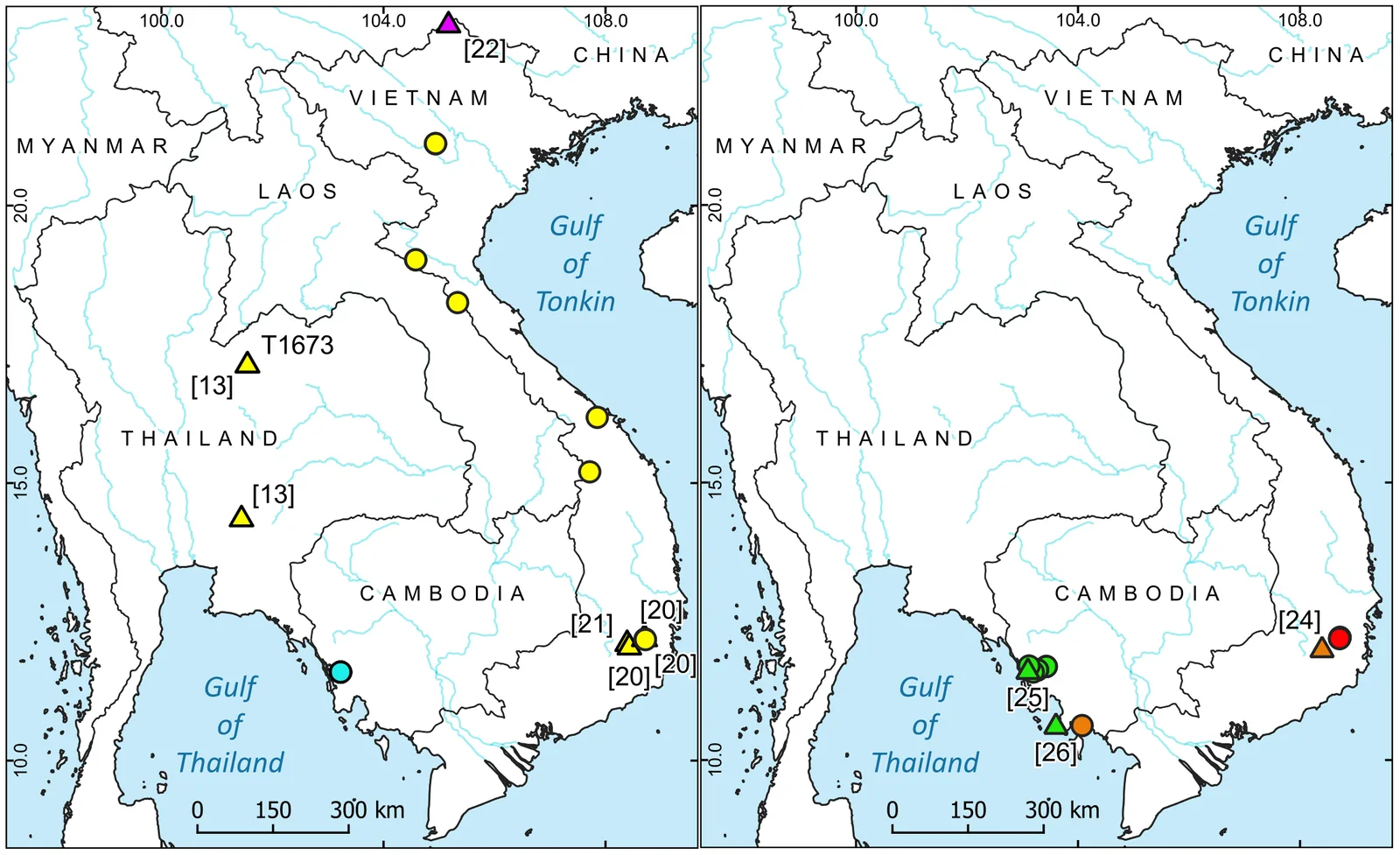
The distribution of *Acromastigum* species in Indochina. Triangles indicate literature records with reference numbers (T1673 refers to an unpublished specimen of *A. herzogii* collected by Tagawa & Kitagawa); circles indicate collecting localities of our specimens. The symbol colors correspond to species as follows: purple — *Acromastigum echinatiforme*, blue—*A. echinatum*, yellow—*A. herzogii*, green—*A. inaequilaterum*, orange—*A. laevigatum*, red—*A. vietnamicum*.

Although our data are incomplete, it is clear that *Acromastigum* is an uncommon participant in plant communities in East Indochina. The northernmost location of the genus is in Hà Giang Province in Northeast Vietnam. *Acromastigum echinatiforme* is reported from there, although we did not examine specimens of this species. A more southern occurrence of the genus belongs to *A. herzogii* (generally the most common species of the genus in Indochina) and comes from Phú Thọ Province in Northeast Vietnam. Further along, the genus occurs quite regularly along the Annamite Range. *Acromastigum herzogii* continues its area to Nghệ An Province, Hà Tĩnh Province, and Hue City Administered Area (formerly Thừa Thiên Huế Province). The main diversity of the genus is observed in the Central Highlands of Vietnam (Quảng Ngãi and Lâm Đồng Provinces). Three species are known from here: *A. laevigatum*, *A. herzogii*, and *A. vietnamicum*. This center of diversity coincides with the area of high taxonomic diversity of *Bazzania* in Vietnam [53], which may indicate similar ecological preferences and distribution patterns of both genera. Another “burst” of diversity is observed in the Cardamom and Elephant Mountains of Cambodia (Kampot and Koh Kong Provinces). Three species are listed from here: *A. laevigatum*, *A. inaequilaterum*, and *A. echinatum*. The data on the *Acromastigum* distribution in Indochina are summarized in Fig 14.

East Indochina is located on the edge of the core area of *Acromastigum*. We find it difficult to determine the climatic or phytogeographic reasons for this. However, given our experience working in East Indochina from 2011 to the present, we can confidently say that *Acromastigum* species are uncommon, if not rare, here. We have never found representatives of this genus with sporophytes, although some taxa were observed with gynoecia and androecia. Perhaps the sexual reproduction of representatives in Indochina is difficult, and the distribution of the genus is correlated with the undisturbed forest communities where these species can survive. In any way, the taxonomic diversity of the genus within Indochina is significantly lower than that along the stretch from Malesia to Australasia. Sixteen species are known in Malaysia [9], 9 in New Guinea, 12 in New Caledonia [2,12], 22 in Australia [3,4], and 8 in New Zealand [54]. Java Island appears to be an incomprehensible gap, where only 2 species of the genus are known [55]. The same maybe true for Philippines where only 4 species are known [56].

In his review of the genus *Acromastigum*, Grolle [11] wrote the following (pp. 254–255, translated from German by the authors): “The new *Acromastigum* species described above not only expand our knowledge of species within the genus *Acromastigum* but also confirm the genus *Acromastigum* as defined by Evans [7] as a natural taxon. While doubts had previously existed as to whether, in particular, the sections *Acromastigum* and *Inaequilatera*, i.e., species with such different habits and stem cross-sections, where different leaf shapes and leaf insertions, as well as differences in the number of capsule layers, can be retained in one genus, there is now hardly any reason to do so”. However, at the present stage, this statement can be called into question. Indeed, according to molecular genetic data, two conditionally isophyllous taxa appear to be in one clade, whereas anisophyllous ones appear in another. Although *A. stellare* is found in a clade with subisophyllous species, its genetic makeup is quite distinct. Moreover, when the data obtained from SplitTree are taken together with the data on *p*-distances (prominent in Table 4), it seems more natural to refer *A. herzogii* to subg. *Inaequilatera*. Morphologically, the species occupies an intermediate position between subisophyllous and anisophyllous species. Since our understanding of the genetic diversity of *Acromastigum* is very limited, we leave this issue outside the scope of our study; however, we believe it is worth highlighting.

In the Results section, we noted that we observed rhizoids formed from apical cells of the scale-lake leaves and underleaves in the ventral flagella in all the studied species of subg. *Inaequilatera*. This feature was not previously mentioned or discussed. Given that we studied only a small portion of the worldwide diversity of this genus, we cannot claim the universality of this feature. However, observations along these lines may be useful in other regions in future research on *Acromastigum*.

## Conclusion

Six species of *Acromastigum* are known from East Indochina. We collected material for five of them. One species, described as new to science, belongs to the species-poor subg. *Acromastigum* and likely represents the northernmost known taxon of this subgenus. Two species are being studied molecularly (including the newly described taxon); the sequences for the other species we collected were not available. The reported distribution of *A. curtilobum* in Thailand is likely based on erroneous identifications and should be replaced with *A. herzogii*.

## Supporting information

S1 File Inclusivity in global research questionnaire. Responses to the inclusivity in global research questionnaire for this study.
(DOCX)

S1 Appendix The list of voucher details and GenBank accession numbers for the specimens used in the phylogenetic analysis in the present paper. The newly obtained sequences are marked in bold.
(DOCX)
