# Supplementary material for: A review of Acromastigum (Lepidoziaceae, Marchantiophyta) in East Indochina
Source: PLoS One. 2026 Sep 3;21(9):e0356719. doi: 10.1371/journal.pone.0356719 (PMC13541187; doi:10.1371/journal.pone.0356719)
Supplement: Supplementary file 2 [file pone.0356719.s002.docx]

Appendix 1. The list of voucher details and GenBank accession numbers for the specimens used in the phylogenetic analysis in the present paper. The newly obtained sequences are marked in bold.

| Accepted name | GenBank name | Label | GenBank Accession Number | | |
| --- | --- | --- | --- | --- | --- |
|  |  |  | *trn*L-*trn*F cpDNA | *rbc*L cpDNA | *trn*G cpDNA |
| *Tricholepidozia chaetocarpa* (Pearson) E.D. Cooper | *Telaranea chaetocarpa* (Pearson) Grolle | New Caledonia, 702433 (NSW) | EF101120 | EF101011 |  |
| *Tricholepidozia lindenbergii* (Gottsche) E.D. Cooper | *Telaranea lindenbergii* (Gottsche) J.J. Engel & G.L. Merr. | New Zealand, J.J. Engel, 23605 (NSW) | JF316608 | JF316316 | JF316570 |
| *Neolepidozia patentissima* (Hook. f. & Taylor) E.D. Cooper | *Telaranea patentissima* (Hook. f. & Taylor) E.A. Hodgs. | Australia, Victoria, 722443 (NSW) | EF101125 | EF101016 |  |
| *Neolepidozia tridactylis* (Lehm. & Lindenb.) E.D. Cooper | *Telaranea tridactylis* (Lehm. & Lindenb.) J.J. Engel & G.L. Merr. | Australia, E.D. Cooper 170 (NSW) | JX289501 | JX289355 |  |
| *Neolepidozia wallichiana* (Gottsche) Fulford & J. Taylor | *Telaranea wallichiana* (Gottsche) R.M. Schust. | Papua New Guinea, E.A. Brown 04/829 (NSW) | JF316609 | JF316328 | JF316561 |
| *Lepidozia laevifolia* (Hook. f. & Taylor) Gottsche, Lindenb. & Nees | *Lepidozia laevifolia* (Hook. f. & Taylor) Gottsche, Lindenb. & Nees | Australia, New South Wales, 700341 (NSW) | EF101109 | EF101000 |  |
| *Lepidozia setigera* Steph. | *Lepidozia setigera* Steph. | New Zealand, J.J. Engel 41 Bog 20 (F) | JF316625 | JF316326 | JF316552 |
| *Lepidozia obtusiloba* Steph. | *Lepidozia obtusiloba* Steph. | Australia, E.D. Cooper 110 (NSW) | JX289513 | JX289398 |  |
| *Kurzia brevicalycina* (Steph.) Grolle | *Kurzia brevicalycina* (Steph.) Grolle | New Caledonia, 611320 (NSW) | EF101102 | EF100993 |  |
| *Kurzia caduciloba* R.M. Schust. | *Kurzia caduciloba* R.M. Schust. | New Caledonia, 611386 (NSW) | EF101103 | EF100994 |  |
| *Kurzia calcarata* (Steph.) Grolle | *Kurzia calcarata* (Steph.) Grolle | New Zealand, E.D. Cooper 330 (NSW) | JF316596 | JF316301 | JF316513 |
| *Kurzia compacta* (Steph.) Grolle | *Kurzia compacta* (Steph.) Grolle | New Zealand, Renner 02102 | EF101104 | EF100995 | JF316512 |
| *Kurzia helophila* R.M. Schust. | *Kurzia helophila* R.M. Schust. | New Zealand, E.D. Cooper 383 (NSW) | JF316597 | JF316300 | JF316515 |
| *Kurzia temnomoides* R.M. Schust. | *Kurzia temnomoides* R.M. Schust. | Australia, New South Wales, 709779 (NSW) | EF101107 | EF100998 |  |
| *Dendrolembidium dendroides* (Carrington & Pearson) Herzog | *Kurzia dendroides* (Carrington & Pearson) Grolle | Australia, New South Wales, 614780 (NSW) | EF101105 | EF100996 |  |
| *Acromastigum vietnamicum* Bakalin, Klimova, Maltseva sp. nov. | *Acromastigum vietnamicum* Bakalin, Klimova, Maltseva sp. nov. | Vietnam, Central Highlands, Lâm Đồng Province, V.A. Bakalin, K.G. Klimova & M.H. Nguyễn, V-69-5-25 (VBGI) | **PZ124676** | **PZ124684** | **PZ124680** |
| *Acromastigum vietnamicum* Bakalin, Klimova, Maltseva sp. nov. | *Acromastigum vietnamicum* Bakalin, Klimova, Maltseva sp. nov. | Vietnam, Central Highlands, Lâm Đồng Province, K.G. Klimova, V.A. Bakalin & M.H. Nguyễn, Viet-80-11-25 (VBGI) | **PZ124677** | **PZ124685** |  |
| *Acromastigum cavifolium* R.M. Schust. | *Acromastigum cavifolium* R.M. Schust. | New Zealand, E.A. Brown 08/278 (NSW) | JF316585 | JF316302 | JF316540 |
| *Acromastigum mooreanum* (Steph.) E.A. Hodgs. | *Acromastigum mooreanum* (Steph.) E.A. Hodgs. | Australia, Tasmania, B. Shaw 6407 (DUKE) | JF316587 | JF316318 | JF316546 |
| *Acromastigum adaptatum* Hürl. | *Acromastigum adaptatum* Hürl. | New Caledonia, E.A. Brown 06/137 (NSW) | JF316589 | JF316320 | JF316541 |
| *Acromastigum colensoanum* (Mitt.) A. Evans ex Reimers | *Acromastigum colensoanum* (Mitt.) A. Evans ex Reimers | Australia, Victoria, 722410 (NSW) | EF101080 | EF100973 |  |
| *Acromastigum exiguum* (Steph.) A. Evans | *Acromastigum exiguum* (Steph.) A. Evans | Australia, New South Wales, 700366 (NSW) | EF101081 | EF100974 |  |
| *Acromastigum filum* (Steph.) A. Evans | *Acromastigum filum* (Steph.) A. Evans | New Caledonia, 611418 (NSW) | EF101082 | EF100975 |  |
| *Acromastigum filum* (Steph.) A. Evans | *Acromastigum filum* (Steph.) A. Evans | New Caledonia, E.A. Brown 03/139 (NSW) |  |  | JF316544 |
| *Acromastigum moratii* N. Kitag. | *Acromastigum moratii* N. Kitag. | New Caledonia, 611651 (NSW) | EF101083 | EF100976 |  |
| *Acromastigum* sp “*furcatifolium”* | *Acromastigum* sp “*furcatifolium”* | Australia, New South Wales, 494476 (NSW) | EF101084 | EF100977 |  |
| *Acromastigum* sp “*furcatifolium”* | *Acromastigum* sp “*furcatifolium”* | Australia, E.A. Brown 02/100 (NSW) |  |  | JF316547 |
| *Acromastigum stellare* N. Kitag. | *Acromastigum stellare* N. Kitag. | New Caledonia, 611419 (NSW) | EF101085 | EF100978 |  |
| *Acromastigum stellare* N. Kitag. | *Acromastigum stellare* N. Kitag. | New Caledonia, E.A. Brown 03/184 (NSW) |  |  | JF316528 |
| *Acromastigum tenax* (Steph.) A. Evans | *Acromastigum tenax* (Steph.) A. Evans | New Caledonia, 701861 (NSW) | EF101086 | EF100979 |  |
| *Zoopsis argentea* (Hook. f. & Taylor) Gottsche, Lindenb. & Nees | *Zoopsis argentea* (Hook. f. & Taylor) Gottsche, Lindenb. & Nees | Australia, Streimann, 1993, 51704 | AY463595 | AY462337 |  |
| *Zoopsis argentea* (Hook. f. & Taylor) Gottsche, Lindenb. & Nees | *Zoopsis argentea* (Hook. f. & Taylor) Gottsche, Lindenb. & Nees | New Zealand, J.J. Engel 23962 (F) |  |  | JF316504 |
| *Acromastigum herzogii* Grolle | *Acromastigum herzogii* Grolle | Vietnam, Northeast Vietnam, Phú Thọ Province, V.A. Bakalin & M.H. Nguyễn, V-21-35-23 (VBGI) | **PZ124678** |  | **PZ124681** |
| *Acromastigum herzogii* Grolle | *Acromastigum herzogii* Grolle | Vietnam, Northeast Vietnam, Phú Thọ Province, V.A. Bakalin & M.H. Nguyễn, V-25-6-23 (VBGI) |  |  | **PZ124682** |
| *Acromastigum herzogii* Grolle | *Acromastigum herzogii* Grolle | Vietnam, North Central Region in North Vietnam, Nghệ An Province, V.A. Bakalin & M.H. Nguyễn, V-37-16-22 (VBGI) | **PZ124679** |  | **PZ124683** |
| *Acromastigum divaricatum* (Nees) A. Evans ex Reimers | *Acromastigum divaricatum* (Nees) A. Evans ex Reimers | Papua New Guinea, E.A. Brown 04/84e (NSW) |  |  | JF316543 |
| *Acromastigum cunninghamii* (Steph.) A. Evans | *Acromastigum cunninghamii* (Steph.) A. Evans | New Zealand, J.J. Engel 23662 (F) |  |  | JF316542 |
| *Acromastigum anisostomum* (Lehm. & Lindenb.) A. Evans | *Acromastigum anisostomum* (Lehm. & Lindenb.) A. Evans | New Zealand, E.C. Davis NZ40 (DUKE) | JF316588 | JF316321 | JF316545 |
